# Incidence of pancreatic cancer is dramatically increased by a high fat, high calorie diet in KrasG12D mice

**DOI:** 10.1371/journal.pone.0184455

**Published:** 2017-09-08

**Authors:** Hui-Hua Chang, Aune Moro, Kazuki Takakura, Hsin-Yuan Su, Allen Mo, Masako Nakanishi, Richard T. Waldron, Samuel W. French, David W. Dawson, O. Joe Hines, Gang Li, Vay Liang W. Go, James Sinnett-Smith, Stephen J. Pandol, Aurelia Lugea, Anna S. Gukovskaya, Michael O. Duff, Daniel W. Rosenberg, Enrique Rozengurt, Guido Eibl

**Affiliations:** 1 Department of Surgery, David Geffen School of Medicine at UCLA, Los Angeles, CA, United States of America; 2 Division of Digestive Diseases, Department of Medicine, David Geffen School of Medicine at UCLA, Los Angeles, CA, United States of America; 3 Veterans Affairs Greater Los Angeles Healthcare System, Los Angeles, CA, United States of America; 4 Pancreatic Research Group, Department of Medicine, Cedars-Sinai Medical Center, Los Angeles, CA, United States of America; 5 Center for Molecular Oncology, UCONN Health, Farmington, CT, United States of America; 6 Department of Pathology, Harbor-UCLA Medical Center, Torrance, CA, United States of America; 7 Southern California Research Center for ALPD and Cirrhosis, Department of Pathology, Keck School of Medicine of the University of Southern California, Los Angeles, CA, United States of America; 8 Department of Pathology and Laboratory Medicine, David Geffen School of Medicine at UCLA, Los Angeles, CA, United States of America; 9 Department of Biostatistics, School of Public Health at UCLA, Los Angeles, CA, United States of America; 10 Department of Genetics and Genome Sciences, UCONN Health, Farmington, CT, United States of America; Vrije Universiteit Brussel, BELGIUM

## Abstract

Epidemiologic data has linked obesity to a higher risk of pancreatic cancer, but the underlying mechanisms are poorly understood. To allow for detailed mechanistic studies in a relevant model mimicking diet-induced obesity and pancreatic cancer, a high-fat, high-calorie diet (HFCD) was given to *P48*^*+/Cre*^*;LSL-KRAS*^*G12D*^ (KC) mice carrying a pancreas-specific oncogenic Kras mutation. The mice were randomly allocated to a HFCD or control diet (CD). Cohorts were sacrificed at 3, 6, and 9 months and tissues were harvested for further analysis. Compared to CD-fed mice, HFCD-fed animals gained significantly more weight. Importantly, the cancer incidence was remarkably increased in HFCD-fed KC mice, particularly in male KC mice. In addition, KC mice fed the HFCD showed more extensive inflammation and fibrosis, and more advanced PanIN lesions in the pancreas, compared to age-matched CD-fed animals. Interestingly, we found that the HFCD reduced autophagic flux in PanIN lesions in KC mice. Further, exome sequencing of isolated murine PanIN lesions identified numerous genetic variants unique to the HFCD. These data underscore the role of sustained inflammation and dysregulated autophagy in diet-induced pancreatic cancer development and suggest that diet-induced genetic alterations may contribute to this process. Our findings provide a better understanding of the mechanisms underlying the obesity-cancer link in males and females, and will facilitate the development of interventions targeting obesity-associated pancreatic cancer.

## Introduction

Pancreatic cancer, of which pancreatic ductal adenocarcinoma (PDAC) represents the vast majority, is a remarkably aggressive and lethal disease with an overall 5-year survival rate of only about 8% [1]. The incidence of this disease in the US is estimated to increase to 53,670 new cases in 2017 and it is currently the fourth leading cause of cancer mortality in both men and women [1]. Despite advances in understanding the tumor biology of pancreatic cancer development, molecularly targeted therapy has not been translated into substantially improved prognosis of this deadly disease. Indeed, total deaths due to pancreatic cancer are projected to increase dramatically to become the second leading cause of cancer-related deaths before 2030 [2]. Consequently, the focus of research has shifted gradually towards its prevention and interception [3–5]. Novel targets and agents for chemo- and/or dietary prevention are urgently needed and will most likely arise from the identification of modifiable risk factors, and from a more detailed understanding of the molecular mechanisms stimulating the promotion and progression of PDAC.

Obesity, which is often attributable to a shift in diet towards increased intake of unhealthy fats, simple carbohydrates, and overall elevated calories, has been positively associated with pancreatic cancer risk [6–10]. Interestingly, visceral obesity (*i*.*e*. excess deposition of intra-abdominal fat) is specifically linked to an increased risk for pancreatic and other cancers, independent of general obesity measured by body mass index (BMI) [9, 11, 12]. Moreover, BMI is imperfect to distinguish between metabolically healthy obesity from metabolically unhealthy obesity with accompanying inflammation and hyperinsulinemia. In view of the compelling epidemiological evidence and the increasing prevalence of obesity, the identification of strategies to disrupt the link between obesity and pancreatic cancer is of central importance. However, the molecular basis underlying this link is complex and still unclear. Several potential mechanisms have been proposed, including metabolic perturbations (hyperinsulinemia, hyperglycemia, or dyslipidemia), unresolved inflammation, and dysregulated autophagy, all of which are common features observed in obesity as well as in pancreatic cancer, creating a tumor-promoting environment [13]. Infiltrating immune cells and stromal elements as components of a robust fibro-inflammatory reaction, along with initiated neoplastic cells, are thought to orchestrate the tumor microenvironment to foster proliferation, survival, metastasis, and immunosuppression, through the production of mediators such as cytokines, chemokines, and prostaglandins [14, 15]. Dysfunctional autophagy has also been linked to pancreatic diseases including pancreatitis and pancreatic cancer [16, 17]. In PDAC development, the function of autophagy seems to be double-edged and context-dependent [18, 19]. It is therefore pertinent to investigate the role of autophagy in obesity-associated PDAC, which remains poorly understood.

In order to provide a platform for detailed mechanistic studies, we have previously described a highly relevant model of diet-promoted pancreatic neoplasia. Specifically, a high-fat, high-calorie diet (HFCD) was given for 3 months to *P48*^*+/Cre*^*;LSL-KRAS*^*G12D*^ (KC) mice carrying a pancreas-specific oncogenic *Kras* mutation [20]. Compared with animals fed a control diet (CD), mice in the HFCD group gained significantly more weight and developed hyperinsulinemia, hyperglycemia, and hyperleptinemia recapitulating conditions associated with the human metabolic syndrome. Importantly, the pancreas of HFCD-fed KC mice showed enhanced inflammation, increased stromal fibrosis, and more advanced PanIN lesions [20]. Furthermore, mice on the HFCD exhibited a significantly increased inflammatory reaction in the visceral adipose tissue (VAT), particularly in the peri-pancreatic fat (PPF), compared to animals on a CD [21]. These results suggest that obesity-associated inflammation in the pancreas and surrounding VAT may accelerate early pancreatic neoplasia in KC mice. However, the effects of the HFCD on the development of invasive pancreatic cancer were not explored. Also, the dynamics of HFCD-promoted tumorigenesis were not addressed in previous studies with short-term and single time-point analysis.

Here, we present further longitudinal and detailed analyses of invasive pancreatic tumor development in KC mice. The effects of the HFCD on inflammatory, fibrotic, and autophagic pathways were investigated. In addition, we performed exome sequencing on isolated advanced PanIN lesions to investigate HFCD-associated genetic alterations. Our data clearly show that diet-induced obesity can promote the development of invasive pancreatic cancer in female and male KC mice providing a suitable and valuable model to further investigate the underlying molecular signals and to evaluate interventions targeting obesity-associated pancreatic cancer development. Furthermore, we described for the first time genetic variants in advanced PanIN lesions that are unique to the HFCD and HFCD-induced obesity, suggesting that diet-induced genetic alterations may underlie and/or contribute to the acceleration of PDAC development by the HFCD.

## Materials and methods

### Experimental animals

The conditional KrasG12D (KC) model of pancreatic neoplasia [22] was used for this study. After weaning, offspring of crosses of *LSL-KRAS*^*G12D*^ and *P48*^*+/Cre*^ mice were randomly allocated to either a control diet (CD) or a high fat, high calorie diet (HFCD). Individually tagged mice had free access to the diet as well as water. Body weights, health and behavior of animals were monitored weekly. Different cohorts of male and female mice were sacrificed at 3, 6, and 9 months of age, and tissues were harvested for histological analysis. Animal studies were approved by the Chancellor’s Animal Research Committee of the University of California, Los Angeles in accordance with the NIH Guide for the Care and Use of Laboratory Animals (protocol number: 2011-118-11). Animals were prematurely euthanized if signs of advanced tumor development (ascites, palpable mass, jaundice, cachexia, weight loss of more than 10%) occurred. None of the animals died without euthanasia.

### Genotyping analysis

Prior to randomization to the study diets, the presence of the *LSL-KRAS*^*G12D*^ and *Cre* alleles in the animals were determined by PCR analysis of genomic DNA, as described elsewhere [23]. Animals with both the *LSL-KRAS*^*G12D*^ and *Cre* alleles were designated as mutant (KC) and animals with neither allele were considered wild-type (WT).

### Experimental diets

The diets were obtained from Dyets, Inc. (Bethlehem, PA). Mice at 1 month of age were randomly allocated to receive either the CD or HFCD. Detailed composition of the diets is shown in **S1 Table**. Briefly, 12% and 40% of calories were derived from fat (corn oil-based) in the CD and HFCD, respectively. The diets were handled under low light conditions, and stored at -20°C for long-term storage or at 4°C for short-term storage in sealed containers. The diets given to mice were replaced weekly.

### Metabolic panel

After blood was collected from the mice by cardiac puncture, plasma was separated by centrifugation at 5,000 rpm for 10 minutes at room temperature, and then stored at −80°C until use. Plasma levels of insulin and leptin were measured using the MILLIPLEX MAP Mouse Adipokine Magnetic Bead Panel—Endocrine Multiplex Assay (EMD Millipore, Billerica, MA) according to the manufacturer's instructions. Blood chemistry (cholesterol, glucose, and triglycerides) was obtained by the UCLA Division of Laboratory Animal Medicine.

### Pancreas histology

Formalin-fixed, paraffin-embedded (FFPE) pancreatic tissues of each animal were sectioned (4 μm) and stained with hematoxylin and eosin (H&E), and were histologically analyzed by a gastrointestinal pathologist blinded to the experimental groups. Inflammation was graded as previously described [20]. Acinar loss was based on the percentage loss across the entire cross-section and graded as 0 = absent; 1 = 1–25%; 2 = 26–50%; 3 = 51–75%; and 4>75%. Inflammation was based on the average number of lobular inflammatory cells per 40x high-power field (HPF; as counted in 10 non-overlapping HPFs) and graded as 0 = absent; 1 = 1–30 cells; 2 = 31–50 cells; 3 = 51–100 cells; and 4>100 cells. Fibrosis was based on the cumulative area of stromal fibrosis across the entire pancreas and graded as 0 = absent; 1 = 1–5%; 2 = 6–10%; 3 = 11–20%; and 4>20%. Murine PanINs and invasive ductal adenocarcinomas were classified according to histopathologic criteria as previously described [24, 25]. The total number of ductal lesions and their grade were determined. Only the highest-grade lesion per pancreatic lobule was evaluated. About 100 pancreatic ducts of the entire fixed specimen (tail of the pancreas) were analyzed for each animal. The relative proportion of each mPanIN to the overall number of analyzed ducts was recorded for each animal.

### Sirius red staining

Sections (4 μm) from FFPE tissues were stained with Sirius red (which stains collagen red) to evaluate the extent of collagen deposition. Whole stained slides were scanned using the AT Turbo slide scanner (Aperio, Vista, CA) and digitized images were visualized using the Leica Digital Image Hub (Leica microsystems). The total fibrosis area stained by Sirius red in each specimen was quantified by morphometric analysis in at least 10 digitized, non-overlapping sections at X200 magnification using the MetaMorph imaging system (Universal Imaging Corporation, PA) and expressed as percentage of total tissue area. The percentage of Sirius red stained area in each specimen was calculated as an average of data obtained from all digitized sections; at least two specimens per mouse were measured, and 3–4 mice per group were investigated.

### Western blotting

Mouse pancreatic tissue samples were homogenized in RIPA (radio-immunoprecipitation assay) buffer containing 50 mmol/L Tris (pH 7.4), 150 mmol/L NaCl, 1% deoxycholic acid, 1% Triton X-100, 0.1% SDS and a mix of protease and phosphatase inhibitors (Roche Applied Science, Basel, Switzerland). Protein extracts were resolved by SDS-PAGE for immunoblot analysis. The following primary antibodies were used: fibronectin (#F3648), prolyl-4-hydroxylase (P4HA; #2SAB1100773), α-SMA (#A2547) and amylase (#A8273) were purchased from Sigma-Aldrich (St. Louis, MO); cadherin 11 (#4442), p-STAT3 (Tyr 705; #9145), and total STAT3 (#9132) from Cell Signaling Technology (Danvers, MA), and glyceraldehyde-3-phosphate dehydrogenase (GAPDH; #9484) from Abcam (Cambridge, UK). Horseradish peroxidase-conjugated specific secondary antibodies were from Cell Signaling Technology (rabbit and mouse). The antibodies were diluted according to the manufacturer's recommendation. Immuno-reactive bands were visualized by chemiluminescence (Pierce) and densitometrically quantified using the PXi Touch Imaging System (Syngene). To estimate protein levels, optical density values in each blot were expressed relative to those of the corresponding loading control.

### Immunofluorescence staining

Immunofluorescence (IF) staining for LC3 and p62 was performed on FFPE pancreas tissue sections. After xylene deparaffinization, ethanol dehydration, and heat-induced antigen retrieval with 0.01 M citrate pH 6.0 was performed, nonspecific binding was blocked with 5% rabbit or 5% goat serum. Tissue sections were incubated with primary antibodies, followed by the incubation with FITC or Texas Red conjugated secondary antibodies. The primary antibody against LC3-B was purchased from Cell Signaling Technology (Danvers, MA), and antibody against p62/SQSTM1 was from Abcam (Cambridge, UK). IF images were acquired with a Zeiss LSM 710 confocal microscope using a 63x objective. Nuclei were stained with DAPI. Differential interference contrast (DIC) was used to display pancreas histology.

### Exome sequencing

Harvested pancreas tissues were embedded in OCT. Serial sections were prepared on PEN slides for laser-capture microdissection (LCM). PanIN-2/3 lesions (identified from flanking H&E slides) were laser-captured from serial sections onto Capsure LCM Caps using an ArcturusXT LCM instrument. Caps were extracted using the PicoPure DNA extraction kit from Thermo Fisher Scientific (Waltham, MA). Extracted DNA was amplified using the SeqPlex DNA Amplification Kit (Sigma-Aldrich, St. Louis, MO) and tested for quality using a Bioanalyzer 2100 and run on a 1% gel prior to library preparation. Sample libraries were prepared using the Swift Biosciences Kit (Accel NGS 2 plus) and Agilent Sure Select XT Mouse Exome post-capture kit following manufacturer's instructions. Sample libraries were pooled and run paired-end (2x75 bp) on an Illumina NextSeq 500 for an average of 90M reads per sample.

### Sequencing data analyses

#### Variant calling, annotation, and prioritization

Sequence data was aligned to the mouse genome (GRCm38 assembly for reference strain C57BL/6J) using the BWA aligner [26]. PCR duplicates were marked and removed, and quality scores of alignments were recalibrated. CD and HFCD reads (n = 4 per group) were pooled together, and VCF (Variant Call Format) files were generated by running Freebayes (Bayesian haplotype-based polymorphism discovery and genotyping: http://arxiv.org/abs/1207.3907) separately on the CD and HFCD read sets with filtering thresholds chosen to be QUAL>10 and DEPTH>30. The set of variants unique to the HFCD group was computed by comparing coordinate and variant information contained in the respective VCF files. VCF files were uploaded to UCSC genome browser (http://genome.ucsc.edu/, [27]) with three tracks: variants found in CD group, HFCD group, and in HFCD but not in CD groups (HF&notCD). Exon coordinates were downloaded from UCSC table browser [28] and reduced to a distinct set of exons per gene (table browser reports identical exons appearing in multiple isoforms). Using Perl scripts, gene symbol IDs were merged into the exon BED file before intersecting with variant positions, and the variant counts for each gene were accumulated from the output of the bedTools intersectBed command [29]. The mouse-specific analysis was executed using freebayes with filtering thresholds set to QUAL>10 & DEPTH>10 (the DEPTH requirement was reduced in this analysis to account for the smaller data set compared to the grouped pooled data). The filtered mouse-specific HFCD VCF files were used to extract the set of variants that did not appear in the CD pooled case, and then they were intersected with exon regions and accumulated as gene-specific hit-counts.

#### Pathway analysis

Pathway analyses were performed using the publicly available database, ConsensusPathDB (CPDB, http://cpdb.molgen.mpg.de/MCPDB). CPDB incorporates data from 16 public databases to generate interaction networks potentially associated with the user-specified list of genes. Genes with at least one variant unique to the HFCD were analyzed by an over-represented pathway analysis. We selected pathways that matched at least 10 genes from the list with the default *p*-value threshold of 0.01. A *p*-value in this software is determined as the result of hypergeometric test based on the number of physical entities present in both the predefined set and user-specified list of physical entities.

### Statistical analysis

Values are presented as mean ± SD or SEM. To determine statistical significance, one-way (or two-way) ANOVA and two-tailed Student’s *t*-tests were performed assuming unequal variances. A *p-*value less than 0.05 was considered significant and was indicated with an asterisk (*).

## Results

### The HFCD accelerates *KRAS*^*G12D*^-driven pancreatic cancer development

**Fig 1:**
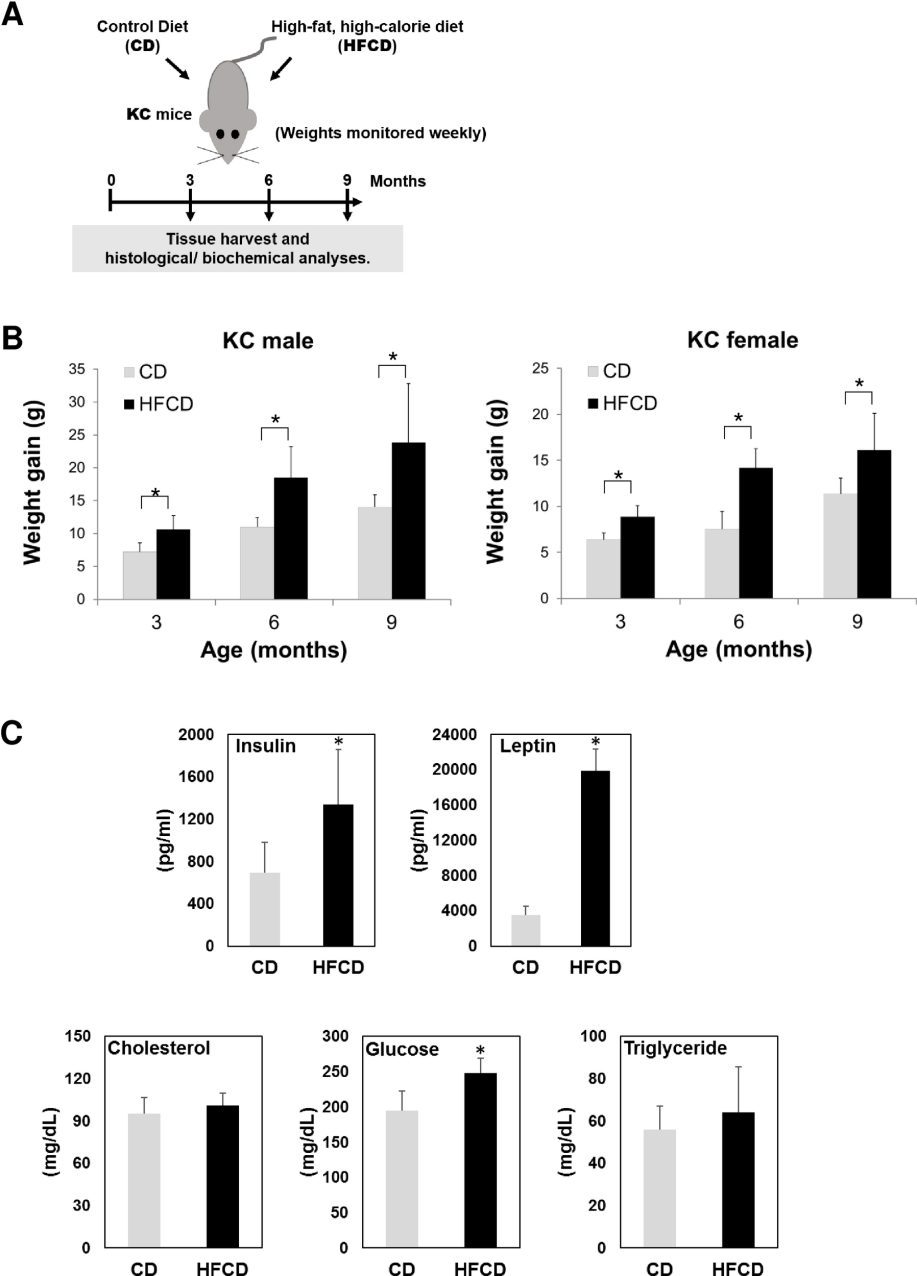
HFCD leads to greater weight gain in male and female KC mice. **(A)** A schematic view of the study design. **(B)** Weight gain of male (left panel) and female (right panel) KC mice fed the CD or HFCD. The values are means ± SD. *P<0.05, Student’s *t*-tests. For the male mice collected at 3, 6, and 9 months, n = 12 (5 on CD and 7 on HFCD), 12 (6 on CD and 6 on HFCD), and 11 (5 on CD and 6 on HFCD), respectively. For the female mice collected at 3, 6, and 9 months, n = 11 (6 on CD and 5 on HFCD), 12 (5 on CD and 7 on HFCD), and 11 (5 on CD and 6 on HFCD), respectively. **(C)** Plasma levels of insulin, leptin, cholesterol, glucose, and triglycerides in 9-month-old KC mice fed the CD or HFCD. Data are depicted as means ± SD. *P<0.05 vs. control, Student’s *t*-tests.

In order to investigate the time-course of pancreatic cancer development promoted by obesity, we used *P48*^*+/Cre*^*;LSL-KRAS*^*G12D*^ (KC) mice carrying a pancreas-specific oncogenic *Kras* mutation [20]. The KC mice were randomly allocated to either the CD or HFCD and were weighed weekly. Different cohorts were sacrificed at 3, 6, and 9 months, and tissues were harvested for further analysis, as illustrated in **Fig 1A**. HFCD-fed KC mice gained more weight (g) than CD-fed KC mice at 3- (9.9 vs. 6.8), 6- (16.2 vs. 9.4), and 9-months (20.0 vs. 12.7) (*p*<0.05 for all ages). Male mice gained more weight than female mice at each time point (*p*<0.05) (**Fig 1B**). We have shown previously that 3-month-old mice fed the HFCD in comparison with CD-fed animals displayed higher levels of insulin, leptin, and glucose in plasma, but there was no significant difference in plasma cholesterol and triglyceride levels between the two diet groups [20]. Here, we observed similar changes in the metabolic panel of the 9-month-old KC mice given CD vs. HFCD (**Fig 1C**).

**Fig 2:**
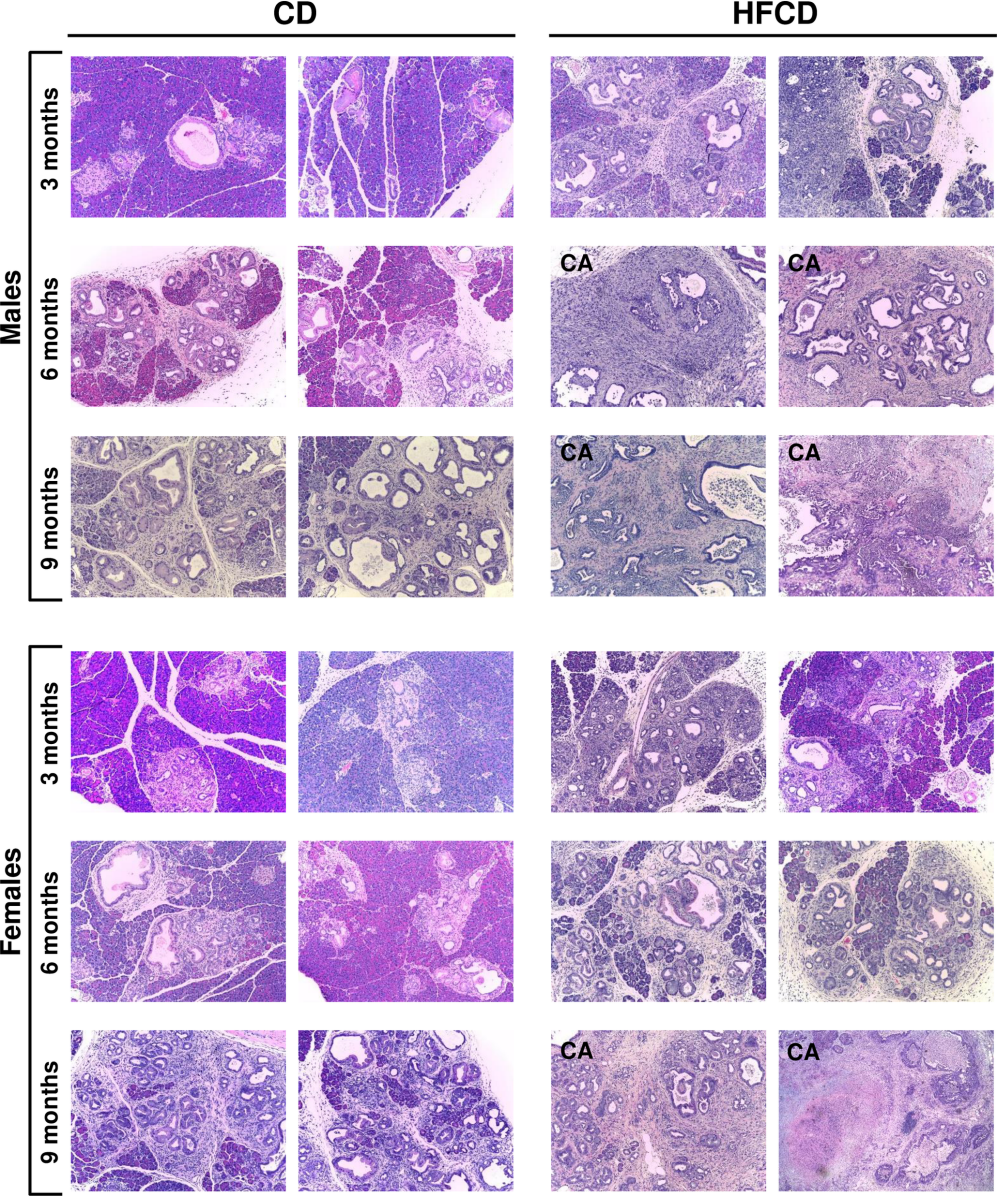
Histological analysis of the pancreas. Pancreas histology of male and female KC mice fed the CD or HFCD for 3, 6, and 9 months. For each group, images (10x) from two different mice were shown. CA, cancer.

**Fig 3:**
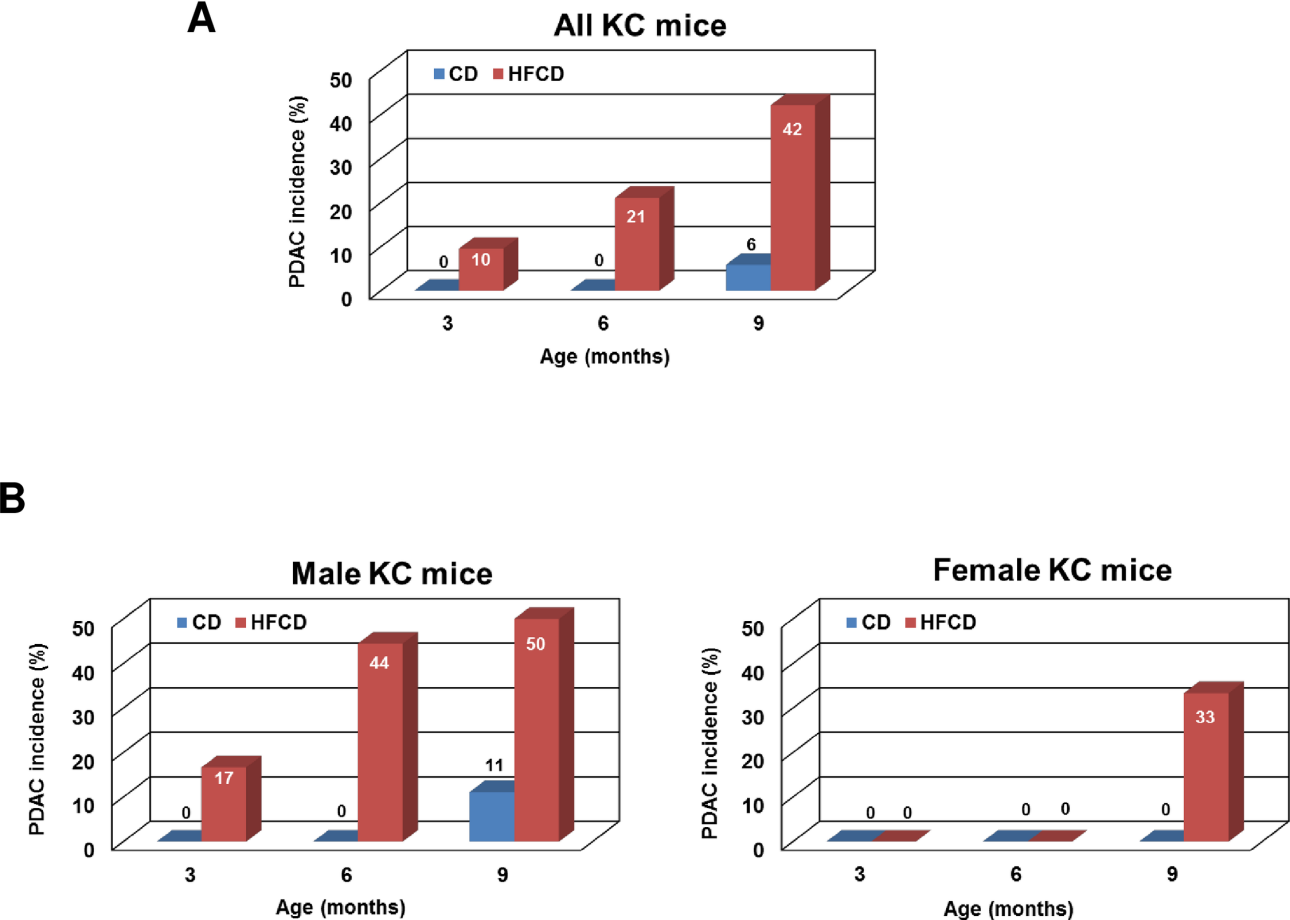
HFCD leads to increased cancer incidence in male and female KC mice. **(A)** The cancer incidence (%) for CD- and HFCD-fed KC mice at indicated ages. **(B)** Gender-specific analyses of the PDAC incidence (%) for CD- and HFCD-fed KC mice at indicated ages. The numbers of mice included in the analyses are: n = 34 for 3-month-old mice including 13 on CD (6 males and 7 females) and 21 on HFCD (12 males and 9 females), n = 31 for 6-month-old mice including 12 on CD (6 males and 6 females) and 19 on HFCD (9 males and 10 females), and n = 36 for 9-month-old mice including 17 on CD (9 males and 8 females) and 19 on HFCD (10 males and 9 females).

A salient feature of the results presented in this study is that the occurrence of pancreatic cancer was remarkably accelerated by the HFCD (**Figs 2** and **3**). The PDAC incidence (%) for CD-fed KC mice was 0 at all ages, and 10, 21, and 42 for HFCD-fed mice at 3, 6, and 9 months, respectively (**Fig 3A**). It is noteworthy that male mice had a higher rate and an earlier onset of malignancy than females (**Fig 3B**). For HFCD-fed KC males, the PDAC incidence (%) was 17, 44, and 50 at 3, 6, and 9 months, respectively (**Fig 3B, left panel**), and 0, 0, and 33 for HFCD-fed KC females (**Fig 3B, right panel**).

**Fig 4:**
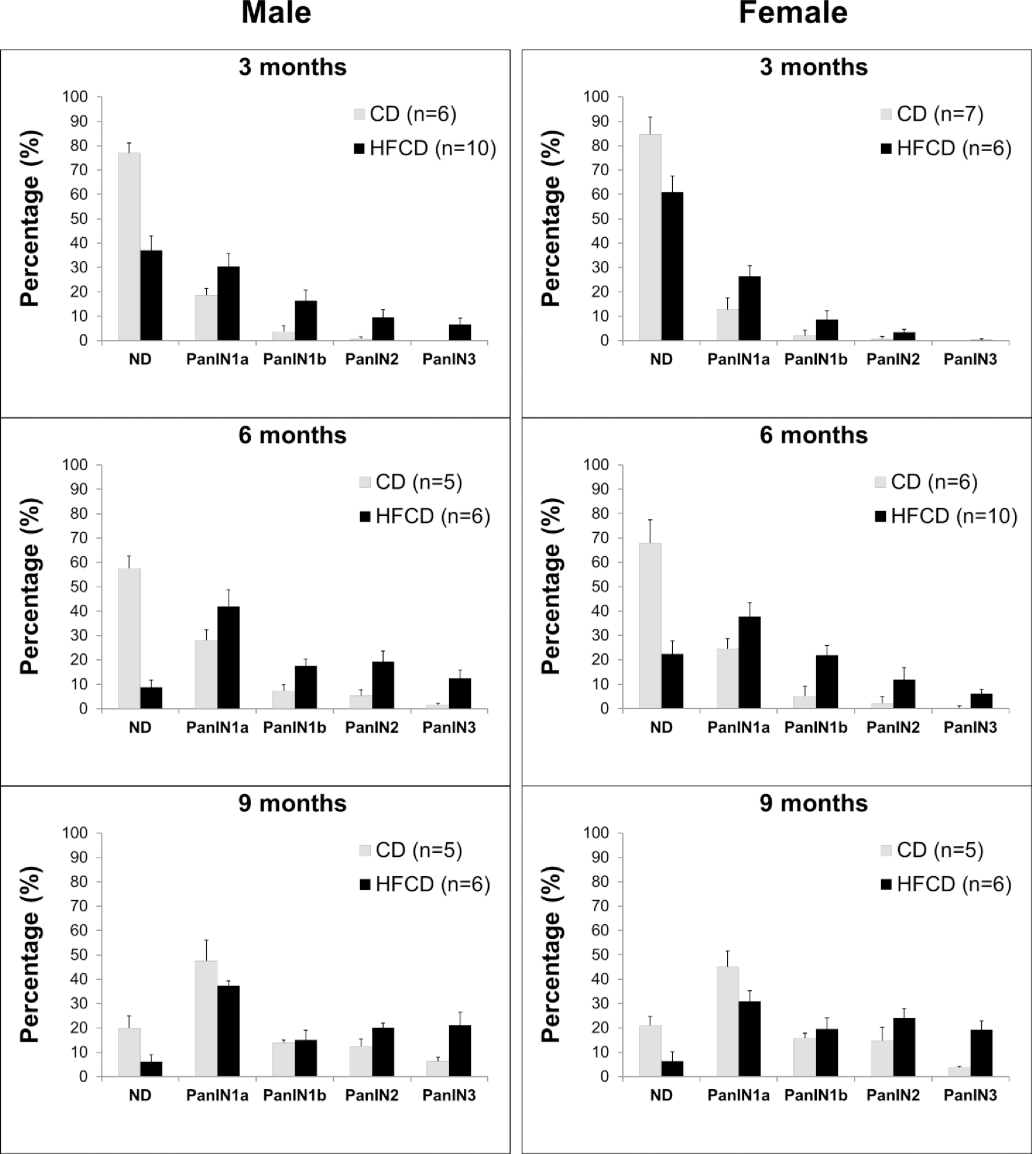
HFCD leads to accelerated pancreatic neoplasia in male and female KC mice. Staged PanIN distribution in cancer-free male and female, CD- and HFCD-fed KC mice at indicated ages. The values are means ± SD.

In addition, KC mice fed the HFCD showed more extensive inflammation and fibrosis, and more advanced PanIN lesions, compared to age-matched CD-fed animals (**Fig 2**). The stage distribution (in %) of PanIN lesions was further evaluated in the pancreas of cancer-free mice. In each age group (3, 6, or 9 months), the KC mice fed the HFCD displayed significantly less normal pancreatic ducts and more advanced PanINs than did those on the CD (**Fig 4**). Overall, these data demonstrate that the HFCD accelerates *KRAS*^*G12D*^-driven PanIN formation and pancreatic cancer development, with a noticeable gender difference.

### The HFCD promotes pancreatic inflammation and fibrosis in KC mice

**Fig 5:**
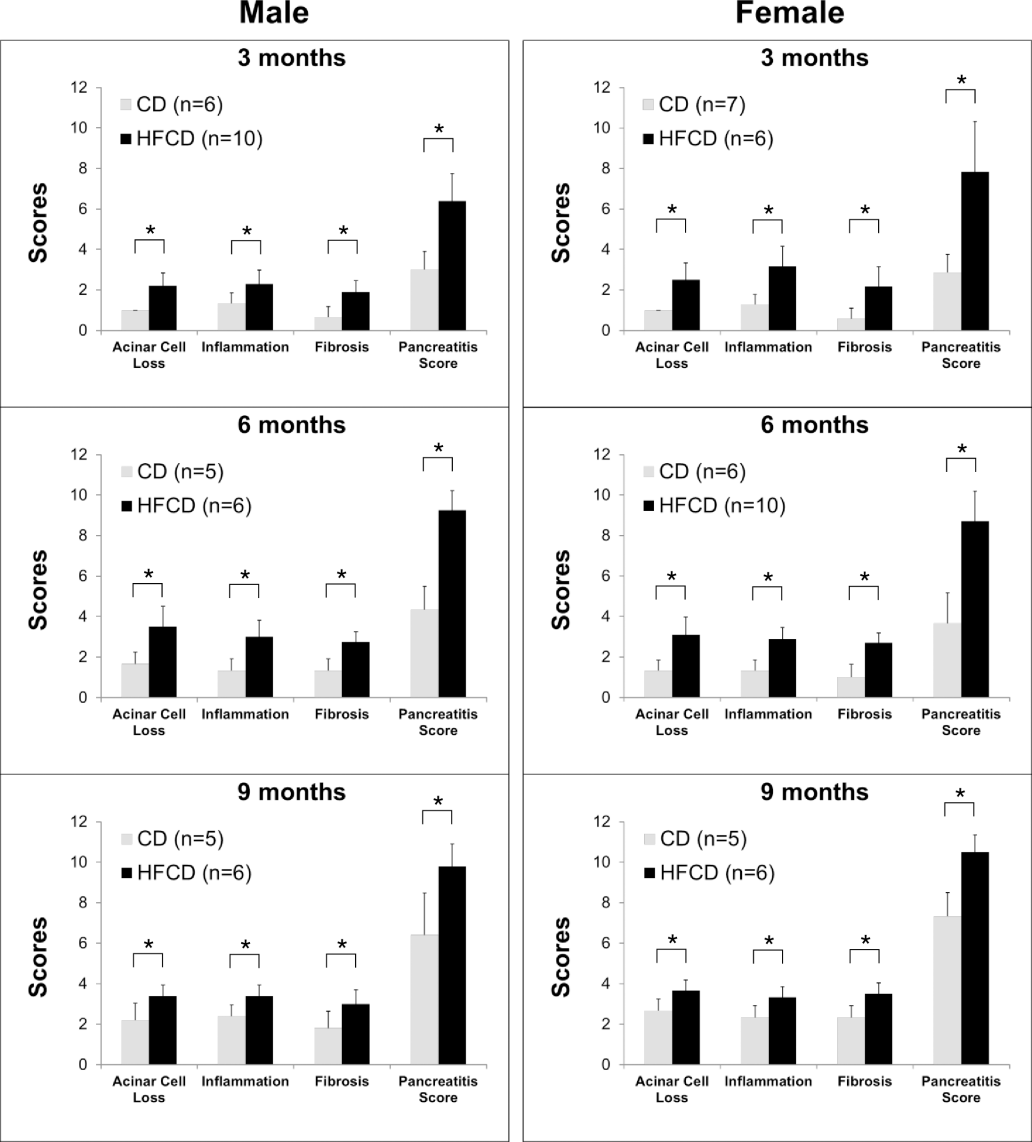
HFCD promotes inflammation in the pancreas of KC mice. Inflammatory parameters in the histological sections of pancreas were evaluated quantitatively. Specifically, acinar loss scores, inflammatory scores, fibrosis scores, and pancreatitis indices were determined for male and female KC mice fed the CD or HFCD for different time periods. The values are means ± SD. *P<0.05, Student’s *t*-tests.

To assess pancreatic inflammation, we histologically evaluated pancreatic tissue sections of KC mice fed the CD and HFCD for different time periods. The pancreatitis index (0–12) was calculated as the sum of individual scores of acinar cell loss, number of infiltrating inflammatory cells, and percentage of stromal fibrosis. Remarkably, scores of these parameters were all significantly greater in the pancreas of HFCD-fed male and female KC mice in every age group (3, 6, or 9 months) (**Fig 5**), indicating that the HFCD leads to more prominent early and sustained pancreatic inflammation.

**Fig 6:**
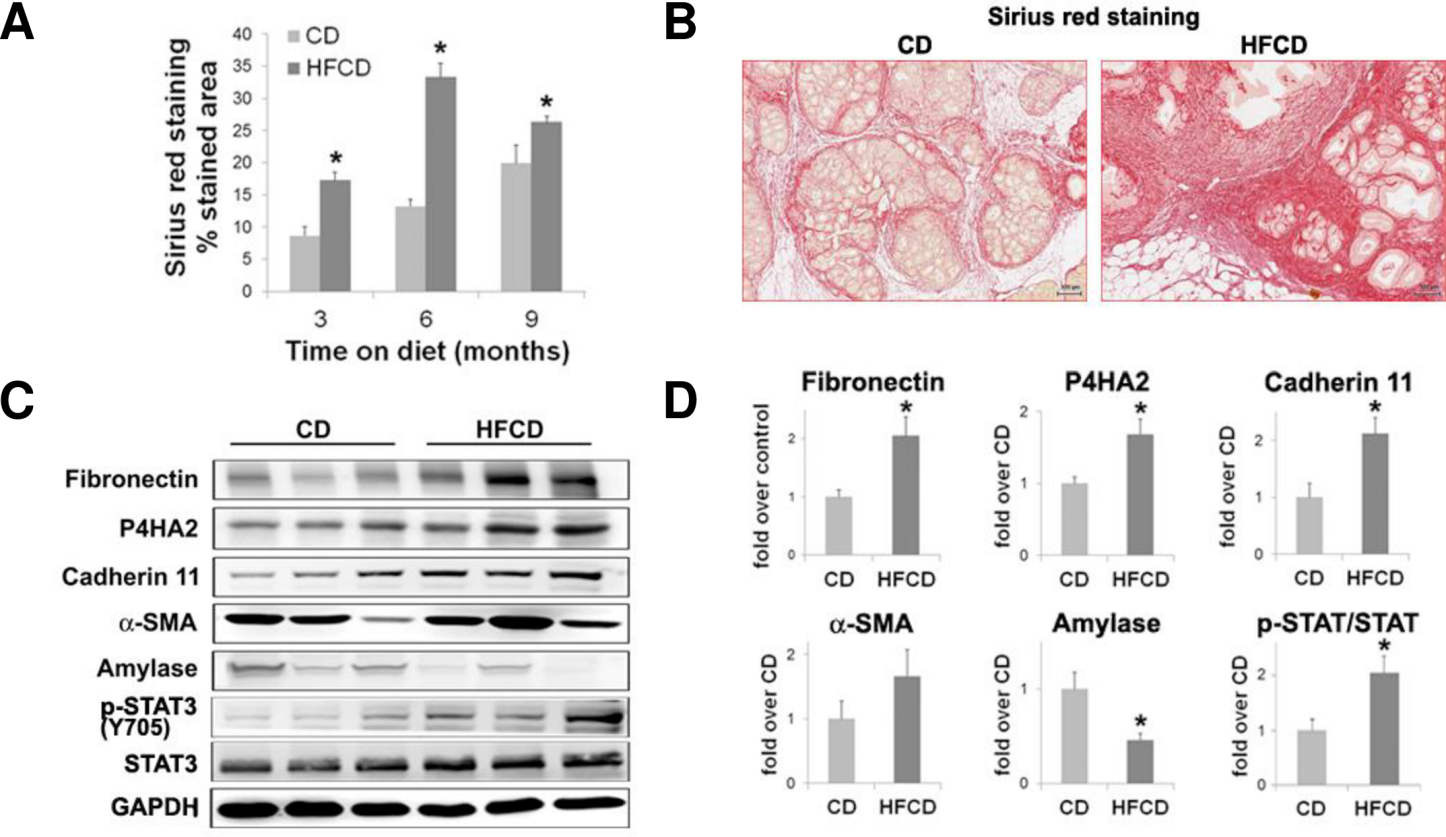
HFCD markedly accelerates stroma formation, extracellular matrix deposition and exocrine atrophy in KC mice. **(A)** The extent of pancreatic collagen deposition was evaluated by Sirius red staining. Graph shows percentage of Sirius red-stained area in pancreas tissue sections at the indicated ages. Data represent mean ± SEM; 8–10 random pancreatic sections were evaluated per mouse; 3–4 mice per group. *P<0.05 vs. CD. **(B)** Pictures illustrate Sirius red staining in pancreatic tissue sections of KC mice fed the CD or HFCD for 6 months, the time-point displaying the highest differences in collagen deposition between CD-fed and HFCD-fed mice. **(C)** Pancreatic levels of fibrosis-related proteins were analyzed by Western blotting in pancreas lysates from KC mice fed the CD or HFCD for 9 months. Picture shows representative immunoblots of fibronectin; prolyl-4-hydroxylase (P4HA2), a key collagen processing enzyme; cadherin 11, a mesenchymal marker expressed by activated myofibroblasts; α-SMA, a myofibroblast marker; and p-STAT3 (Y705)/ total STAT3. Picture also shows protein levels of pancreatic amylase, a digestive enzyme produced by acinar cells and GAPDH used as loading control. Each lane represents an individual mouse; three mice per group are shown. **(D)** Graphs show optical density of immunoblots depicted in panel D. Data in graphs represent mean ± SEM, n = 3. *P<0.05 vs. CD.

Inflammation in the neoplastic pancreas is associated with stromal fibrosis, which is thought to be regulated by pancreatic stellate cells [30]. Characteristics of a fibrotic response include increased production of stromal elements such as collagen and fibronectin. Pancreatic tissues from KC mice fed the HFCD were highly fibrotic (**Fig 6**). As determined by Sirius red staining, the levels of collagen deposition were significantly greater in the pancreas of KC mice fed the HFCD for 3, 6 and 9 months (**Fig 6A** and **6B**). At 9 months, these mice display thicker collagen bundles around PanINs than KC mice fed CD (**Fig 6B**), suggesting increased ECM stiffness. In addition, the HFCD led to the upregulation of fibronectin, Prolyl 4-Hydroxylase, Alpha Polypeptide II (P4HA2, a key component of the enzyme prolyl 4-hydroxylase that is required for the formation of 4-hydroxyproline and folding of newly synthesized collagen fibers), cadherin 11 (a type II cadherin expressed by mesenchymal cells that regulates stroma remodeling, cell migration and metastasis; [31, 32]) and alpha smooth muscle actin (α-SMA, a marker of activated pancreatic stellate cells in the pancreas (**Fig 6C** and **6D**). Furthermore, HFCD induced in KC mice marked activation of signal transducer and activator of transcription-3 (STAT3), as indicated by increase phosphorylation of STAT3 (Y705) in HFCD-fed compared to CD-fed mice (**Fig 6D**). STAT3 is a key regulator of pancreatic cancer development and progression [33, 34] and recent evidence indicates that these effects are mediated by STAT3-induced stroma remodeling and increased ECM stiffness [35]. In accordance with the histological data showing acinar cell loss, levels of amylase were markedly decreased in the pancreas of HFCD-fed mice (**Fig 6C and 6D**). Together, these data demonstrate that HFCD promotes an enhanced fibro-inflammatory reaction in the pancreas of KC mice.

### The HFCD reduced autophagic flux in PanIN lesions of KC mice

**Fig 7:**
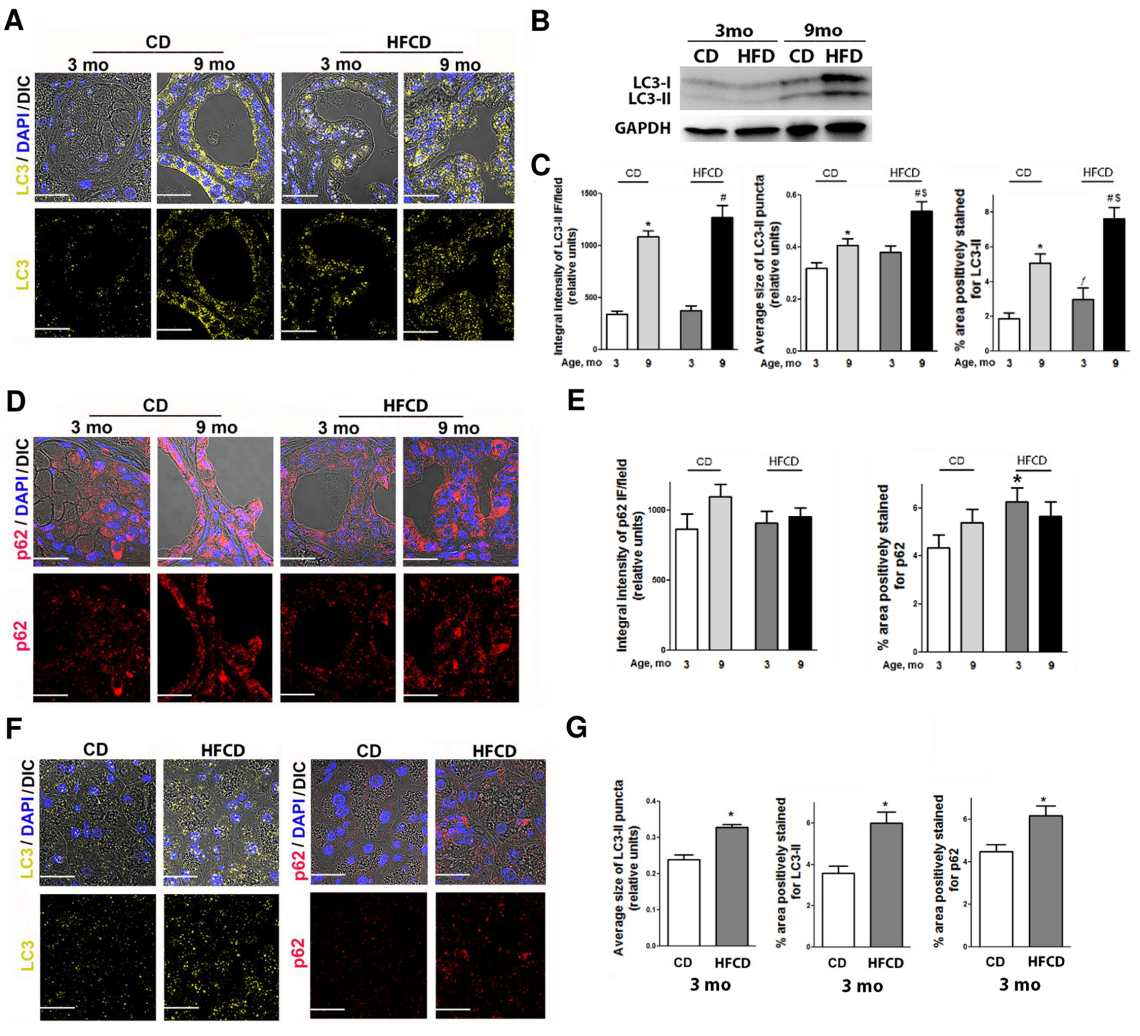
HFCD leads to an accumulation of autophagic vacuoles and p62/SQSTM1. **(A,D,F)** Representative IF images of LC3-II **(A,F)**, p62/SQSTM1 (p62) **(D,F)** in PanIN lesions **(A,D)**, and histologically normal exocrine pancreas **(F)** of KC mice of indicated age fed CD and high fat calorie diet HFCD. DAPI was used to stain nuclei, and DIC to visualize tissue structure (upper lines). Scale bar: 20 μm. **(B)** Immunoblot analysis of LC3 in pancreas from 3- and 9-month-old KC mice. GAPDH is a loading control. **(C,E,G)** Quantification of LC3-II **(C,G)** and p62 **(E,G)** integral intensity, average size of puncta and % positively stained area was performed with ImageJ software. The values are means ± SEM (3 mice were analyzed for each strain, age and diet). * *P*< 0.05 vs. 3 months (mo) CD, # *p* < 0.0001 vs. 3 mo HFCD, $ *p* < 0.05 vs. 9 mo CD, ƒ *p*< 0.05 vs. 3 months (mo) CD.

Autophagy fuels malignant cells under certain conditions (*e*.*g*. metabolic stress caused by desmoplasia) and is known to stimulate progression of PDAC in a KRAS model [36]. To investigate the role of autophagy in obesity-associated pancreatic cancer, we therefore examined the effects of the HFCD on autophagy in the pancreas of KC mice at different ages (3 or 9 months) utilizing immunofluorescence (IF)-based and immunoblot (IB)-methods. As shown in **Fig 7A and 7B**, PanIN lesions in KC mice on either diet showed an age-dependent accumulation of autophagic vacuoles evidenced by the accumulation of LC3-II puncta for IF and the intensity of LC3 for IB [37]. Notably, the HFCD potentiated vacuole accumulation in PanIN lesions as manifested by an increase in the integral intensity of LC3-II puncta, average size of LC3-II dots, and area occupied by LC3-II, with the effect being more pronounced at 9 month of age (**Fig 7C**).

In addition to LC3-II, we also measured the changes in p62, a multifunctional protumorigenic adaptor protein accumulated in human PDAC, known to stimulate the proinflammatory and anti-apoptotic transcription factor NF-kB and promote cancer development [38–40]. Immunostaining of p62, which accumulates with ubiquitin-conjugated aggregates, has been recommended as a histochemical diagnostic marker for protein aggregates [41]. Furthermore, p62 is specifically degraded by autophagy and is widely used as an indicator of autophagic flux efficiency [31]. Interestingly, PanIN lesions do not reveal any significant changes in p62 integral intensity with age or diet (**Fig 7D and 7E**). However, the HFCD increased % area positively stained for p62, indicating accumulation of protein aggregates (**Fig 7E**). It is noteworthy that the HFCD promoted formation of autophagic vacuoles and p62-labeled protein aggregates not only in PanINs but also in morphologically normal areas of pancreas from KC mice (**Fig 7F and 7G**). Taken together, these data suggest that the HFCD leads to inefficient (dysregulated) autophagy in the pancreas of KC mice.

### Exome sequencing revealed genetic variants unique to the HFCD

In order to investigate whether the HFCD or the HFCD-induced inflammatory microenvironment induces genetic alteration that could explain the acceleration of PDAC development in HFCD-fed KC mice, we performed exome sequencing on laser-captured PanIN-2/3 lesions of CD- and HFCD-fed KC mice. Exome sequencing identified a total of 4,856 variants in microdissected PanIN-2/3 lesions specific to the pooled HFCD group. These variants were then processed to generate a list of somatically mutated genes with a read-depth of 30 and a quality score of 10. A total of 2,986 genes were found to contain at least a single variant call (**S2 Table**). The number of variants unique to the HFCD group ranged from 1 to 36 per gene, with the majority of the genes having only a single variant (75%). As expected, the KrasG12D mutation was detected in each of the pancreatic lesions, confirming the validity of the exome sequencing. There were no additional somatic mutations present within the Kras gene. Moreover, there were no somatic mutations detected in a panel of genes that are commonly mutated in human PDAC, including *Tp53*, *Smad4* and *Ink4a/ Arf*.

Using this gene set, we used the CPDB analysis tool to determine whether the constellation of mutated genes may be involved in specific signaling pathways. We identified 33 signaling pathways that were uniquely altered by the HFCD, in which at least 10 genes from the list were involved. Based on *p*-values, the top five pathways are listed in **Table 1**. Many of the variants that are specific to the HFCD group were identified in genes involved in several key pathways: transport of small molecules between cells and the microenvironment, including a solute carrier (SLC) group of membrane transport proteins and ECM components (**Table 1**).

**Table 1:** Enriched pathways of genes with variants unique to HFCD (pooled analysis).

Pathways	# of genes in the pathway	# of genes from the list	*p*-value
**Transmembrane transport of small molecules**	499	100 (20.2%)	1.97E-07
**SLC-mediated transmembrane transport**	249	53 (21.3%)	3.38E-05
**ECM-receptor interaction—Mus musculus (mouse)**	87	24 (27.6%)	8.48E-05
**Transport of glucose and other sugars, bile salts and organic acids, metal ions and amine compounds**	95	25 (26.3%)	0.00014
**Amine compound SLC transporters**	30	11 (36.7%)	0.000534

To identify pathways that are common among individual mice, we generated a list of genes with HFCD-specific variants for each mouse. The number of genes with one or more variants ranged from 1,203 to 1,827 between mice with filters set for a read-depth of 10 and a quality score of 10. Genes that were specifically altered within the HFCD group were similar among mice (in particular mice #HF_S5, HF_S7, and HF_S8), confirming a remarkably consistent effect of the HFCD on inducing genetic alterations (**Table 2**). Using the lists of genes, mouse-specific CPDB analyses were performed as described above. The result showed that several pathways among all four HFCD-fed KC mice were commonly altered, including transmembrane transport, PI3K-Akt signaling, insulin signaling, ECM-receptor interaction, SLC-mediated transmembrane transport and G protein signaling pathways (**Table 3**).

**Table 2:** List of genes and variant counts unique to HFCD in individual mice.

HF_S4	Count	HF_S5	Count	HF_S7	Count	HF_S8	Count
Mroh2a	12	Klra22	35	Klra22	35	Klra22	35
Hjurp	10	BC048546	34	BC048546	34	BC048546	34
Col6a6	9	Klra6	28	Klra6	28	Polr1a	30
Vmn2r121	8	Klra18	25	Klra18	25	Urb1	29
Muc4	7	Polr1a	25	Polr1a	25	Klra6	28
Ccp110	6	Urb1	25	Urb1	25	Klra18	25
Ddo	6	Klra7	24	Klra7	24	Klra7	24
Gm13152	6	Klra9	20	Klra9	20	Klra9	20
Ppwd1	6	Klra5	19	Klra5	19	Klra5	19
Tmc5	6	Mpo	17	Mroh2a	18	Mroh2a	19
Col6a4	5	Ankrd26	15	Mpo	17	Muc4	19
Col6a5	5	Ints10	15	Muc4	17	Ankrd26	17
Pkd1l2	5	Mroh2a	14	Ankrd26	15	Hjurp	17
Slc22a16	5	Muc4	14	Hjurp	15	Mpo	17
Cdh1	4	Dnah6	13	Ints10	15	Ptcd3	17
Gm2022	4	Hjurp	13	Dnah6	13	Ints10	15
Parp8	4	Klra1	13	Klra1	13	Dnah6	14
Smg1	4	Klra17	13	Klra17	13	Als2	13
Smim15	4	Ptcd3	13	Ptcd3	13	Gm2022	13
Tmc7	4	Pzp	12	Als2	12	Klra1	13
Tyk2	4	Unc13a	12	Pzp	12	Klra17	13
Ugt1a6a	4	Als2	11	Unc13a	12	Pzp	12
Vmn2r117	4	Ankfn1	11	Vmn2r121	12	Unc13a	12
9030624J02Rik	3	Itpr1	11	Ankfn1	11	Vmn2r121	12
C130026I21Rik	3	Lpl	11	Col6a6	11	Ankfn1	11
Cct4	3	Mks1	11	Immt	11	Col6a6	11
Coq7	3	Olfr462	11	Itpr1	11	Immt	11
Ddx4	3	Rfxank	11	Lpl	11	Itpr1	11
Dux	3	Col6a6	10	Mks1	11	Lpl	11
Elovl7	3	Immt	10	Olfr462	11	Mks1	11
Ipo11	3	Klra12	10	Rfxank	11	Olfr462	11
Olfr1532-ps1	3	Klrb1c	10	Klra12	10	Rfxank	11
Olfr207	3	Vmn2r121	10	Klrb1c	10	Klra12	10
Olfr611	3	1700030K09Rik	9	Prmt9	10	Klrb1c	10
Ppfibp1	3	Ano2	9	1700030K09Rik	9	Prmt9	10
Trim23	3	Klrb1a	9	Ano2	9	Synj1	10

**Table 3:** Enriched pathways and genes (≥10 genes) with variants common to each HFCD-fed mouse.

Pathways	HFCD-specific genes with variants	# of genes
Transmembrane transport of small molecules	Ano8; Tpcn1; Tpcn2; Nup107; Slc22a16; Ano6; Ano2; Slco3a1; Slc18a1; Slc6a13; Slc6a11; Abcd2; Slc22a8; Slc8a3; Atp4a; Atp2a3; Atp10a; Gnas; Trpc1; Gng2; Nup155; Clcnka; Heph; Atp6v1b2; Atp2c1; Slc25a10; Adcy5; Slc2a4; Slc22a21; Wnk1; Atp8b1; Mcoln3; Trpa1; Abcc6; Gabra1; Cftr; Abcc3; Slc6a2; Aaas; Adcy7; Slc7a9; Slc27a1; Nup54; Atp1b2; Unc80; Slc26a1; Slc22a7; Abcg1; Slc5a5; Slc5a7; Slc15a2; Nup210	52
PI3K-Akt signaling pathway	Nos3; Fn1; Itga4; Pkn3; Hgf; Ifnar1; Lpar2; Rps6kb2; Lpar4; Itgb3; Comp; Col5a2; Col5a3; Insr; Gng2; Mtor; Lamb3; Flt1; Cdkn1b; Igf1r; Col27a1; Pik3r2; Col6a3; Col6a4; Col6a6; Itga9; Pdgfra; Lama4; Chad; Il7; Itga2b; Rps6kb1; Akt2; Chrm1; Itga7; Jak2; Jak3; Fgf23; Kras	39
Insulin Signaling	Snap25; Rac2; Kif5b; Pik3c2a; Map3k14; Igf1r; Map4k2; Insr; Map2k6; Slc2a4; Prkcd; Map3k4; Pfkm; Pik3r4; Stxbp4; Egr1; Rps6kb1; Akt2; Cbl; Rps6kb2; Ptprf; Mtor; Pik3c2g; Pik3r2	24
SLC-mediated transmembrane transport	Nup107; Slc22a16; Slc5a11; Slc22a3; Slc6a13; Slc18a2; Slc24a1; Slc9a1; Slc44a4; Nup133; Nup98; Slc16a1; Slc9a4; Slco3a1; Slc10a6; Slc2a9; Slc28a1; Slc2a2; Slc7a11; Slco1a1; Slc12a3; Gm14085; Slc15a1; Slc4a8	24
ECM-receptor interaction	Itga9; Itgb3; Lamb3; Lama4; Fn1; Chad; Comp; Itga4; Itga7; Col5a2; Col5a3; Col27a1; Itga2b; Agrn; Col6a3; Col6a4; Col6a6	17
Cholinergic synapse	Adcy5; Adcy7; Plcb4; Camk2d; Chrm1; Cacna1c; Akt2; Camk2b; Chrna6; Jak2; Pik3r2; Slc5a7; Itpr3; Itpr1; Gnai2; Gng2; Kras	17
Estrogen signaling pathway	Adcy5; Nos3; Plcb4; Gnas; Sp1; Hspa8; Prkcd; Fkbp4; Fkbp5; Akt2; Adcy7; Pik3r2; Itpr3; Itpr1; Gnai2; Kras	16
Dilated cardiomyopathy	Itga9; Itgb3; Adcy7; Dmd; Myh7; Adcy5; Itga2b; Cacna1c; Itga4; Itga7; Mybpc3; Ttn; Myh6; Gnas; Cacna2d4	15
G alpha (s) signalling events	Adcy5; Ghrhr; Pde4c; Gnas; Ptger4; Crhr2; Drd5; Pth2r; Pde2a; Adcy7; Gng2; Gipr; Adcyap1r1; Gnai2; Ghrh	15
G Protein Signaling Pathways	Adcy5; Adcy7; Akap1; Gnas; Itpr1; Prkcd; Akap9; Akap5; Gna13; Pde4c; Gnai2; Kras	12

Given the known importance of the insulin (PI3K/Akt/mTORC1) signaling pathway in nutrient sensing and PDAC development [42], we analyzed in detail the HFCD-induced genetic alterations within this pathway. We found several mutations in key molecules within the insulin signaling pathway, *e*.*g*. mTOR and class II PI3K isoforms that may be of functional relevance. For example, we detected a non-synonymous substitution T2345A in exon 51 of mTOR that occurred at a frequency of 50%. This mutation causes an amino acid change from a methionine (A**T**G) to lysine (A**A**G) in position 2,345, resulting in a change in polarity (non-polar to polar), pH (neutral to basic) and hydropathy to the protein. Most importantly, this codon is located within the kinase domain of the protein, suggesting possible changes in kinase activity.

## Discussion

There is strong epidemiologic evidence linking obesity to an increased risk of cancer, including pancreatic cancer [10] but the mechanisms involved have remained incompletely understood. The results presented in the current study provide a comprehensive time-course of pancreatic cancer development in female and male mice and for the first time analyzes genetic exomic alterations in PanIN lesions in HFCD-fed obese KC mice. This detailed analysis identified several major and novel observations:

1. The most salient finding of the current study is a substantially increased incidence of invasive pancreatic cancer in KC mice fed the HFCD. This result is in line with a very recent report showing that a high fat diet significantly promotes primary pancreatic cancer growth and the rate of metastasis in the KPC mouse model (with additional *p53* mutation) [43]. In our study, the development of PDAC was associated with more extensive pancreatic inflammation and fibrosis, and dysregulated autophagy in the pancreas. Although the mechanisms driving the obesity-cancer link are not fully understood, a number of factors are implicated, such as the pro-inflammatory state and altered levels of adipokines associated with excess adiposity [44]. Additionally, in obesity there are elevated levels of growth hormones (*i*.*e*. insulin and insulin like growth factor-1) as a consequence of the insulin resistance, which is also causally linked to dysfunctional adipose tissues, especially VATs [45]. Our previous and current findings reinforce the importance of an obesity-associated inflammatory environment in diet-promoted PanIN development and cancer formation, as enhanced inflammation was observed in the pancreas of the HFCD-fed mice ([20] and **Fig 5**). Importantly, we recently showed that KC mice on the HFCD exhibited a significantly more robust inflammation in the VATs, particularly in the depot adjacent and overlying the pancreas (peri-pancreatic), compared to animals on the CD [21]. These results suggest that VAT inflammation is a critical promoting factor in obesity-promoted neoplastic progression.
2. Surprisingly, our results revealed a strong gender difference, with male KC mice displaying a significantly higher rate and earlier onset of pancreatic cancer in response to the HFCD compared to female KC mice (**Fig 3B**). Although the elucidation of the precise mechanism(s) requires further experimental work, our findings suggest a tumor-protecting role of estrogens. Interestingly, estrogens are thought to be protective against VAT gain [46], which may also underlie the increased risk of obesity-associated metabolic disturbances in men. The human data are supported by our animal findings demonstrating a greater weight gain (**Fig 1B**) and a significantly greater expansion of the VAT in male mice as shown previously [20]. In addition, since pancreatic cancer usually occurs in older patients, the putative protective effect of estrogens may disappear in post-menopausal women, which may explain the only slightly increased risk of pancreatic cancer in men [1].
3. Another unexpected finding of our study is that despite a dramatic increase in pancreatic cancer incidence in male (compared to female) KC mice, no significant difference in HFCD-associated pancreatic inflammation between male and female KC mice was observed. This was particularly evident in the 6-month cohort. Although pancreatic inflammation was assessed histologically and subtler differences in inflammatory processes, including distinct cytokine production and/or infiltrating immune cells cannot be rule out, these findings suggest that the different pancreatic cancer incidence between male and female KC mice is independent of HFCD-associated pancreatic inflammation. However, the finding that the HFCD (compared to the CD) led to an increase in the pancreatic inflammatory score as well as to more advanced PanIN lesions similarly in males and females suggests a promoting effect of the HFCD and HFCD-associated pancreatic inflammation in early stages of pancreatic neoplasia.
4. Our data also demonstrated dysregulated autophagy in PanIN lesions of KC mice fed the HFCD, as evident by an accumulation of p62 and increase in LC3-II indicating reduced autophagy flux. It has been shown that a high fat diet alters membrane lipid content to reduce autophagosome/lysosome fusion in the liver, thus suppressing the autophagic flux [47]. Together, these effects may cause an increase in autophagic vacuoles concomitant with reduced autophagic flux. In PanIN lesions from the HFCD-fed KC mice we indeed found accumulation of autophagic vacuoles without a corresponding decrease in p62-labeled protein aggregates. Accumulation of these aggregates may represent one mechanism, by which autophagy dysregulation promotes tumorigenesis in obesity. Although the precise contribution and role of autophagy in pancreatic cancer is complex, recent evidence clearly indicate a tumor-promoting role of autophagy in pancreatic cancer [36, 48]. In contrast, our data show increased incidence of pancreatic cancer in KC mice fed the HFCD despite inefficient autophagy. This interesting observation may suggest that the HFCD overcomes or circumvents a reduction in the tumor-promoting effects of autophagy, possibly by directly providing required nutrients for tumor cell growth independent of autophagy.
5. It is plausible that HFCD-induced obesity with accompanied inflammatory changes in the pancreatic microenvironment causes additional genetic alterations that can accelerate PDAC development. Exome sequencing on laser-captured PanIN-2/3 lesions of KC mice fed the CD or HFCD revealed 4,856 genetic variants (in 2,986 genes) that were unique to the HFCD. Interestingly, we found a remarkable consistency in HFCD-induced genetic variants in 3 out of 4 KC mice. Overall, our data suggest that genetic alterations (mutations) may underlie or contribute to the tumor-promoting effect of the HFCD and HFCD-induced obesity; however, further analyses are clearly needed to determine the functional consequence of these variants. Pathway analysis has identified common networks that were uniquely altered by the HFCD, including the insulin and PI3K/Akt pathway. Given the importance of these pathways in nutrient sensing and pancreatic cancer development [42], we have further analyzed HFCD-induced genetic variants in genes within these networks. Several mutations with possible functional significance were detected. For example, we found mutations (unique to the HFCD) in the kinase domain of mTOR, which may modulate its activity. However, detailed *in vitro* experiments are needed to clearly determine the functional significance of these mutations.

In conclusion, our current study identifies several novel and provocative aspects of diet-induced and obesity-associated pancreatic cancer development in the conditional KrasG12D mouse model. These include a marked increase in invasive pancreatic cancer incidence, profound gender differences, a disconnection between pancreatic inflammation and gender-specific cancer incidence, dysregulated autophagy despite increased cancer formation, and genetic alterations unique to the HFCD in key signaling pathways. Our results will greatly stimulate further studies investigating the precise mechanisms involved and open up experimental avenues for identifying novel aspects in the prevention of PDAC.

## Supporting information

S1 Table Composition of the experimental diets. (DOCX)

S2 Table List of genetic variants (≥10 variant counts) unique to HFCD-fed mice (pooled analysis). (DOCX)

S1 Files Original data for Fig 1 and Figs 3–7. (ZIP)

S2 Files Original data for the exome sequencing. (ZIP)
