# Supplementary material for: Incidence of pancreatic cancer is dramatically increased by a high fat, high calorie diet in KrasG12D mice
Source: PLoS One. 2017 Sep 8;12(9):e0184455. doi: 10.1371/journal.pone.0184455 (PMC5590955; doi:10.1371/journal.pone.0184455)
Supplement: S1 Table — (DOCX) [file pone.0184455.s001.docx]

**S1 Table.** Composition of the experimental diets.

|  | **AIN-76A (CD)** | | |  | **HFCD** | | |
| --- | --- | --- | --- | --- | --- | --- | --- |
|  | **g/kg** | **kcal/g** | **kcal/kg** |  | **g/kg** | **kcal/g** | **kcal/kg** |
| **Corn oil** | 50 | 9 | 450 |  | 200 | 9 | 1,800 |
| **Cornstarch** | 269.2 | 3.6 | 969.12 |  | 119.2 | 3.6 | 429.12 |
| **Casein** | 200 | 3.58 | 716 |  | 200 | 3.58 | 716 |
| **Sucrose** | 380.8 | 4 | 1,523.2 |  | 380.8 | 4 | 1,523.2 |
| **Cellulose** | 50 | 0 | 0 |  | 50 | 0 | 0 |
| **DL-Methionine** | 3 | 4 | 12 |  | 3 | 4 | 12 |
| **Salt mix** | 35 | 0.47 | 16.45 |  | 35 | 0.47 | 16.45 |
| **Vitamin mix** | 10 | 3.92 | 39.2 |  | 10 | 3.92 | 39.2 |
| **Choline bitartrate** | 2 | 0 | 0 |  | 2 | 0 | 0 |
| **Total** |  |  | 3,725.97 |  |  |  | 4,535.97 |
