# Supplementary material for: Incidence of pancreatic cancer is dramatically increased by a high fat, high calorie diet in KrasG12D mice
Source: PLoS One. 2017 Sep 8;12(9):e0184455. doi: 10.1371/journal.pone.0184455 (PMC5590955; doi:10.1371/journal.pone.0184455)
Supplement: S2 Table — (DOCX) [file pone.0184455.s002.docx]

**S2 Table.** List of genetic variants (≥10 variant counts) unique to HFCD-fed mice (pooled analysis).

| **Gene** | | **Variant** |
| --- | --- | --- |
| **Symbol** | **Description** | **Count** |
| **Polr1a** | polymerase (RNA) I polypeptide A | 36 |
| **BC048546** | protein that interacts with Mir proteins | 35 |
| **Klra22** | killer cell lectin-like receptor subfamily A, member 22 | 34 |
| **Urb1** | ribosome biogenesis 1 homolog | 29 |
| **Klra6** | killer cell lectin-like receptor subfamily A, member 6 | 26 |
| **Klra18** | killer cell lectin-like receptor subfamily A, member 18 | 25 |
| **Klra7** | killer cell lectin-like receptor subfamily A, member 7 | 25 |
| **Klra9** | killer cell lectin-like receptor subfamily A, member 9 | 20 |
| **Mpo** | myeloperoxidase | 19 |
| **Hjurp** | Holliday junction recognition protein | 18 |
| **Klra5** | killer cell lectin-like receptor subfamily A, member 5 | 18 |
| **Ankrd26** | ankyrin repeat domain 26 | 17 |
| **Muc4** | mucin 4 | 17 |
| **Ptcd3** | pentatricopeptide repeat domain 3 | 17 |
| **Ints10** | integrator complex subunit 10 | 15 |
| **Dnah6** | dynein, axonemal, heavy chain 6 | 13 |
| **Klra1** | killer cell lectin-like receptor subfamily A, member 1 | 13 |
| **Klra17** | killer cell lectin-like receptor subfamily A, member 17 | 13 |
| **Unc13a** | unc-13 homolog A | 13 |
| **Itpr1** | inositol 1,4,5-trisphosphate receptor 1 | 12 |
| **Mroh2a** | maestro heat-like repeat family member 2A | 12 |
| **Pzp** | pregnancy zone protein | 12 |
| **Rfxank** | regulatory factor X-associated ankyrin-containing protein | 12 |
| **Tmem100** | transmembrane protein 100 | 12 |
| **Als2** | amyotrophic lateral sclerosis 2 | 11 |
| **Ankfn1** | ankyrin-repeat and fibronectin type III domain containing 1 | 11 |
| **Immt** | inner membrane protein, mitochondrial | 11 |
| **Lpl** | lipoprotein lipase | 11 |
| **Mks1** | Meckel syndrome, type 1 | 11 |
| **Olfr462** | olfactory receptor 462 | 11 |
| **Col6a6** | collagen, type VI, alpha 6 | 10 |
| **Synj1** | synaptojanin 1 | 10 |
