# Supplementary material for: Incidence of pancreatic cancer is dramatically increased by a high fat, high calorie diet in KrasG12D mice
Source: PLoS One. 2017 Sep 8;12(9):e0184455. doi: 10.1371/journal.pone.0184455 (PMC5590955; doi:10.1371/journal.pone.0184455)
Supplement: S1 Files — (ZIP) [file pone.0184455.s003.zip › Fig6/Fig6A/12months_t-test.pdf]

t-test Thursday, May 25, 2017, 12:06:26

Data source: sirius red in Notebook

Normality Test: Passed (P > 0.200)

Equal Variance Test: Passed (P = 0.918)

**Group Name**	**N**	**Missing**	**Mean**	**Std Dev**	**SEM**
12M-CD	12	0	28.990	4.630	1.337
12M-HF	19	0	26.228	4.606	1.057

Difference 2.762

t = 1.623 with 29 degrees of freedom. (P = 0.115)

95 percent confidence interval for difference of means: -0.718 to 6.243

The difference in the mean values of the two groups is not great enough to reject the possibility that the difference is due to random sampling variability. There is not a statistically significant difference between the i nput groups (P = 0.115).

Power of performed test with alpha = 0.050: 0.221

The power of the performed test (0.221) is below the desired power of 0.800.
You should interpret the negative findings cautiously.
