# Supplementary material for: Incidence of pancreatic cancer is dramatically increased by a high fat, high calorie diet in KrasG12D mice
Source: PLoS One. 2017 Sep 8;12(9):e0184455. doi: 10.1371/journal.pone.0184455 (PMC5590955; doi:10.1371/journal.pone.0184455)
Supplement: S1 Files — (ZIP) [file pone.0184455.s003.zip › Fig6/Fig6A/3months_t-test.pdf]

t-test Thursday, May 25, 2017, 12:03:06

Data source: sirius red in Notebook

Normality Test: Passed (P > 0.200)

Equal Variance Test: Failed (P = 0.003)

**Group Name**	**N**	**Missing**	**Mean**	**Std Dev**	**SEM**
3M-CD	21	0	8.637	5.890	1.285
3M-HF	16	0	17.820	10.713	2.678

Difference -9.182

t = -3.331 with 35 degrees of freedom. (P = 0.002)

95 percent confidence interval for difference of means: -14.779 to -3.586

The difference in the mean values of the two groups is greater than would be expected by chance; there is a statistically significant difference between the input groups (P = 0.002).

Power of performed test with alpha = 0.050: 0.888
