# Supplementary material for: Incidence of pancreatic cancer is dramatically increased by a high fat, high calorie diet in KrasG12D mice
Source: PLoS One. 2017 Sep 8;12(9):e0184455. doi: 10.1371/journal.pone.0184455 (PMC5590955; doi:10.1371/journal.pone.0184455)
Supplement: S1 Files — (ZIP) [file pone.0184455.s003.zip › Fig6/Fig6A/6months_t-test.pdf]

t-test Thursday, May 25, 2017, 12:04:18

Data source: sirius red in Notebook

Normality Test: Passed (P > 0.200)

Equal Variance Test: Passed (P = 0.854)

**Group Name**	**N**	**Missing**	**Mean**	**Std Dev**	**SEM**
6M-CD	22	0	13.445	7.215	1.538
6M-HF	27	0	33.332	7.163	1.379

Difference -19.887

t = -9.635 with 47 degrees of freedom. (P = <0.001)

95 percent confidence interval for difference of means: -24.040 to -15.735

The difference in the mean values of the two groups is greater than would be expected by chance; there is a statistically significant difference between the input groups (P = <0.001).

Power of performed test with alpha = 0.050: 1.000
