# Supplementary material for: Incidence of pancreatic cancer is dramatically increased by a high fat, high calorie diet in KrasG12D mice
Source: PLoS One. 2017 Sep 8;12(9):e0184455. doi: 10.1371/journal.pone.0184455 (PMC5590955; doi:10.1371/journal.pone.0184455)
Supplement: S1 Files — (ZIP) [file pone.0184455.s003.zip › Fig6/Fig6A/9months_t-test.pdf]

t-test Thursday, May 25, 2017, 12:05:36

Data source: sirius red in Notebook

Normality Test: Passed (P > 0.200)

Equal Variance Test: Passed (P = 0.493)

Group Name	N	Missing	Mean	Std Dev	SEM
9M-CD	18	0	20.750	5.204	1.227
9M-HF	22	0	26.228	6.272	1.337

Difference -5.478

t = -2.962 with 38 degrees of freedom. (P = 0.005)

95 percent confidence interval for difference of means: -9.221 to -1.734

The difference in the mean values of the two groups is greater than would be expected by chance; there is a statistically significant difference between the input groups (P = 0.005).

Power of performed test with alpha = 0.050: 0.788

The power of the performed test (0.788) is below the desired power of 0.800.
You should interpret the negative findings cautiously.
