# Supplementary material for: Incidence of pancreatic cancer is dramatically increased by a high fat, high calorie diet in KrasG12D mice
Source: PLoS One. 2017 Sep 8;12(9):e0184455. doi: 10.1371/journal.pone.0184455 (PMC5590955; doi:10.1371/journal.pone.0184455)
Supplement: S1 Files — (ZIP) [file pone.0184455.s003.zip › Fig6/Fig6A/ANOVA_Sirius_red.pdf]

Two Way Analysis of Variance Thursday, May 25, 2017, 11:58:45

Data source: sirius red in Notebook

General Linear Model

Dependent Variable: % stained area

Normality Test: Passed (P > 0.200)

Equal Variance Test: Failed (P = 0.003)

**Source of Variation**	**DF**	**SS**	**MS**	**F**	**P**
time	3	3896.757	1298.919	28.686	<0.001
diet	1	2351.785	2351.785	51.938	<0.001
time x diet	3	2573.671	857.890	18.946	<0.001
Residual	149	6746.856	45.281		
Total	156	17200.886	110.262		

The difference in the mean values among the different levels of time is greater than would be expected by chance after allowing for effects of differences in diet. There is a statistically significant difference (P = <0.001). To isolate which group(s) differ from the others use a multiple comparison procedure.

The difference in the mean values among the different levels of diet is greater than would be expected by chance after allowing for effects of differences in time. There is a statistically significant difference (P = <0.001). To isolate which group(s) differ from the others use a multiple comparison procedure.

The effect of different levels of time depends on what level of diet is present. There is a statistically significant interaction between time and diet. (P = <0.001)

Power of performed test with alpha = 0.0500: for time : 1.000
Power of performed test with alpha = 0.0500: for diet : 1.000
Power of performed test with alpha = 0.0500: for time x diet : 1.000

Least square means for time :

**Group**	**Mean**	**SEM**
3M	13.228	1.117
6M	23.389	0.966
9M	23.489	1.069
12M	27.609	1.241

Least square means for diet :

**Group**	**Mean**	**SEM**
CD	17.956	0.810
HF	25.902	0.748

Least square means for time x diet :

**Group**	**Mean**	**SEM**
3M x CD	8.637	1.468
3M x HF	17.820	1.682
6M x CD	13.445	1.435
6M x HF	33.332	1.295
9M x CD	20.750	1.586

9M x HF	26.228	1.435	12M x CD	28.990	1.943
12M x HF	26.228	1.544			

All Pairwise Multiple Comparison Procedures (Tukey Test):

Comparisons for factor: **time**

**Comparison**	**Diff of Means**	**p**	**q**	**P**	**P<0.050**
12M vs. 3M	14.380	4	12.185	<0.001	Yes
12M vs. 6M	4.220	4	3.795	0.037	Yes
12M vs. 9M	4.120	4	3.557	0.058	No
9M vs. 3M	10.260	4	9.386	<0.001	Yes
9M vs. 6M	0.100	4	0.0984	1.000	No
6M vs. 3M	10.160	4	9.731	<0.001	Yes

Comparisons for factor: **diet**

**Comparison**	**Diff of Means**	**p**	**q**	**P**	**P<0.050**
HF vs. CD	7.946	2	10.192	<0.001	Yes

Comparisons for factor: **diet within 3M**

**Comparison**	**Diff of Means**	**p**	**q**	**P**	**P<0.05**
HF vs. CD	9.182	2	5.815	<0.001	Yes

Comparisons for factor: **diet within 6M**

**Comparison**	**Diff of Means**	**p**	**q**	**P**	**P<0.05**
HF vs. CD	19.887	2	14.552	<0.001	Yes

Comparisons for factor: **diet within 9M**

**Comparison**	**Diff of Means**	**p**	**q**	**P**	**P<0.05**
HF vs. CD	5.478	2	3.622	0.010	Yes

Comparisons for factor: **diet within 12M**

**Comparison**	**Diff of Means**	**p**	**q**	**P**	**P<0.05**
CD vs. HF	2.762	2	1.574	0.266	No

Comparisons for factor: **time within CD**

**Comparison**	**Diff of Means**	**p**	**q**	**P**	**P<0.05**
12M vs. 3M	20.353	4	11.820	<0.001	Yes
12M vs. 6M	15.545	4	9.104	<0.001	Yes
12M vs. 9M	8.240	4	4.647	0.006	Yes
9M vs. 3M	12.113	4	7.925	<0.001	Yes
9M vs. 6M	7.305	4	4.831	0.004	Yes
6M vs. 3M	4.808	4	3.312	0.089	No

Comparisons for factor: **time within HF**

**Comparison**	**Diff of Means**	**p**	**q**	**P**	**P<0.05**
6M vs. 3M	15.513	4	10.334	<0.001	Yes

6M vs. 9M	7.105	4	5.199	0.001	Yes
6M vs. 12M	7.105	4	4.986	0.002	Yes
12M vs. 3M	8.408	4	5.208	0.001	Yes
12M vs. 9M	0.00000957	4	0.00000642	1.000	No
9M vs. 3M	8.408	4	5.378	<0.001	Yes
