# Supplementary material for: Incidence of pancreatic cancer is dramatically increased by a high fat, high calorie diet in KrasG12D mice
Source: PLoS One. 2017 Sep 8;12(9):e0184455. doi: 10.1371/journal.pone.0184455 (PMC5590955; doi:10.1371/journal.pone.0184455)
Supplement: S1 Files — (ZIP) [file pone.0184455.s003.zip › Fig6/Fig6A/ANOVA_sirius_red_means.pdf]

Two Way Analysis of Variance Thursday, May 25, 2017, 12:10:30

Data source: sirius red in Notebook

General Linear Model

Dependent Variable: Col 13

Normality Test: Passed (P > 0.200)

Equal Variance Test: Passed (P = 0.049)

**Source of Variation**	**DF**	**SS**	**MS**	**F**	**P**
time2	3	632.236	210.745	30.420	<0.001
diet2	1	402.507	402.507	58.100	<0.001
time2 x diet2	3	340.063	113.354	16.362	<0.001
Residual	16	110.845	6.928		
Total	23	1545.989	67.217		

The difference in the mean values among the different levels of time2 is greater than would be expected by chance after allowing for effects of differences in diet2. There is a statistically significant difference (P = <0.001). To isolate which group(s) differ from the others use a multiple comparison procedure.

The difference in the mean values among the different levels of diet2 is greater than would be expected by chance after allowing for effects of differences in time2. There is a statistically significant difference (P = <0.001). To isolate which group(s) differ from the others use a multiple comparison procedure.

The effect of different levels of time2 depends on what level of diet2 is present. There is a statistically significant interaction between time2 and diet2. (P = <0.001)

Power of performed test with alpha = 0.0500: for time2 : 1.000
Power of performed test with alpha = 0.0500: for diet2 : 1.000
Power of performed test with alpha = 0.0500: for time2 x diet2 : 1.000

Least square means for time2 :

**Group**	**Mean**	**SEM**
3M	12.952	1.075
6M	23.265	1.075
9M	23.127	1.005
12M	27.222	1.201

Least square means for diet2 :

**Group**	**Mean**	**SEM**
CD	17.482	0.806
HF	25.800	0.736

Least square means for time2 x diet2 :

**Group**	**Mean**	**SEM**
3M x CD	8.637	1.520
3M x HF	17.267	1.520
6M x CD	13.200	1.520
6M x HF	33.330	1.520
9M x CD	19.913	1.520

9M x HF	26.340	1.316	12M x CD	28.180	1.861
12M x HF	26.263	1.520			

All Pairwise Multiple Comparison Procedures (Tukey Test):

Comparisons for factor: **time2**

**Comparison**	**Diff of Means**	**p**	**q**	**P**	**P<0.050**
12M vs. 3M	14.270	4	12.521	<0.001	Yes
12M vs. 9M	4.095	4	3.697	0.080	No
12M vs. 6M	3.957	4	3.472	0.106	Do Not Test
6M vs. 3M	10.313	4	9.598	<0.001	Yes
6M vs. 9M	0.138	4	0.133	1.000	Do Not Test
9M vs. 3M	10.175	4	9.780	<0.001	Yes

Comparisons for factor: **diet2**

**Comparison**	**Diff of Means**	**p**	**q**	**P**	**P<0.050**
HF vs. CD	8.317	2	10.780	<0.001	Yes

Comparisons for factor: **diet2 within 3M**

**Comparison**	**Diff of Means**	**p**	**q**	**P**	**P<0.05**
HF vs. CD	8.630	2	5.679	0.001	Yes

Comparisons for factor: **diet2 within 6M**

**Comparison**	**Diff of Means**	**p**	**q**	**P**	**P<0.05**
HF vs. CD	20.130	2	13.247	<0.001	Yes

Comparisons for factor: **diet2 within 9M**

**Comparison**	**Diff of Means**	**p**	**q**	**P**	**P<0.05**
HF vs. CD	6.427	2	4.521	0.006	Yes

Comparisons for factor: **diet2 within 12M**

**Comparison**	**Diff of Means**	**p**	**q**	**P**	**P<0.05**
CD vs. HF	1.917	2	1.128	0.437	No

Comparisons for factor: **time2 within CD**

**Comparison**	**Diff of Means**	**p**	**q**	**P**	**P<0.05**
12M vs. 3M	19.543	4	11.503	<0.001	Yes
12M vs. 6M	14.980	4	8.817	<0.001	Yes
12M vs. 9M	8.267	4	4.866	0.016	Yes
9M vs. 3M	11.277	4	7.421	<0.001	Yes
9M vs. 6M	6.713	4	4.418	0.030	Yes
6M vs. 3M	4.563	4	3.003	0.188	No

Comparisons for factor: **time2 within HF**

**Comparison**	**Diff of Means**	**p**	**q**	**P**	**P<0.05**
6M vs. 3M	16.063	4	10.571	<0.001	Yes

6M vs. 12M	7.067	4	4.650	0.022	Yes
6M vs. 9M	6.990	4	4.917	0.015	Yes
9M vs. 3M	9.073	4	6.383	0.002	Yes
9M vs. 12M	0.0767	4	0.0539	1.000	No
12M vs. 3M	8.997	4	5.920	0.004	Yes

A result of "Do Not Test" occurs for a comparison when no significant difference is found between two means that enclose that comparison. For example, if you had four means sorted in order, and found no difference between means 4 vs. 2, then you would not test 4 vs. 3 and 3 vs. 2, but still test 4 vs. 1 and 3 vs. 1 (4 vs. 3 and 3 vs. 2 are enclosed by 4 vs. 2: 4 3 2 1). Note that not testing the enclosed means is a procedural rule, and a result of Do Not Test should be treated as if there is no significant difference between the means, even though one may appear to exist.
