# Supplementary material for: Incidence of pancreatic cancer is dramatically increased by a high fat, high calorie diet in KrasG12D mice
Source: PLoS One. 2017 Sep 8;12(9):e0184455. doi: 10.1371/journal.pone.0184455 (PMC5590955; doi:10.1371/journal.pone.0184455)
Supplement: S1 Files — (ZIP) [file pone.0184455.s003.zip › Fig6/Fig6D/Amylase_OD_t-test.pdf]

t-test Thursday, May 25, 2017, 18:35:28

Data source: western OD in Notebook

Normality Test: Passed (P > 0.200)

Equal Variance Test: Passed (P = 0.514)

**Group Name**	**N**	**Missing**	**Mean**	**Std Dev**	**SEM**
Amylase_CD	3	0	1.000	0.305	0.176
Amylase_HF	3	0	0.461	0.115	0.0664

Difference 0.539

t = 2.866 with 4 degrees of freedom. (P = 0.046)

95 percent confidence interval for difference of means: 0.0168 to 1.062

The difference in the mean values of the two groups is greater than would be expected by chance; there is a statistically significant difference between the input groups (P = 0.046).

Power of performed test with alpha = 0.050: 0.530

The power of the performed test (0.530) is below the desired power of 0.800.
You should interpret the negative findings cautiously.
