# Supplementary material for: Incidence of pancreatic cancer is dramatically increased by a high fat, high calorie diet in KrasG12D mice
Source: PLoS One. 2017 Sep 8;12(9):e0184455. doi: 10.1371/journal.pone.0184455 (PMC5590955; doi:10.1371/journal.pone.0184455)
Supplement: S1 Files — (ZIP) [file pone.0184455.s003.zip › Fig6/Fig6D/aSMA_OD_t-test.pdf]

t-test Thursday, May 25, 2017, 18:36:09

Data source: western OD in Notebook

Normality Test: Passed (P > 0.200)

Equal Variance Test: Passed (P = 0.676)

Group Name	N	Missing	Mean	Std Dev	SEM
SMA_CD	3	0	1.000	0.484	0.280
SMA_HF	3	0	1.663	0.709	0.410

Difference -0.663

t = -1.336 with 4 degrees of freedom. (P = 0.252)

95 percent confidence interval for difference of means: -2.039 to 0.714

The difference in the mean values of the two groups is not great enough to reject the possibility that the difference is due to random sampling variability. There is not a statistically significant difference between the i nput groups (P = 0.252).

Power of performed test with alpha = 0.050: 0.105

The power of the performed test (0.105) is below the desired power of 0.800.
You should interpret the negative findings cautiously.
