# Supplementary material for: Incidence of pancreatic cancer is dramatically increased by a high fat, high calorie diet in KrasG12D mice
Source: PLoS One. 2017 Sep 8;12(9):e0184455. doi: 10.1371/journal.pone.0184455 (PMC5590955; doi:10.1371/journal.pone.0184455)
Supplement: S1 Files — (ZIP) [file pone.0184455.s003.zip › Fig6/Fig6D/Fibronectin_OD_t-test.pdf]

t-test Thursday, May 25, 2017, 15:54:05

Data source: western OD in Notebook

Normality Test: Passed (P > 0.200)

Equal Variance Test: Passed (P = 0.274)

**Group Name**	**N**	**Missing**	**Mean**	**Std Dev**	**SEM**
Fibro_CD	3	0	1.003	0.211	0.122
Fibro_HF	3	0	2.057	0.565	0.326

Difference -1.053

t = -3.024 with 4 degrees of freedom. (P = 0.039)

95 percent confidence interval for difference of means: -2.021 to -0.0861

The difference in the mean values of the two groups is greater than would be expected by chance; there is a statistically significant difference between the input groups (P = 0.039).

Power of performed test with alpha = 0.050: 0.579

The power of the performed test (0.579) is below the desired power of 0.800.
You should interpret the negative findings cautiously.
