# Supplementary figures and images for: Incidence of pancreatic cancer is dramatically increased by a high fat, high calorie diet in KrasG12D mice
Source: PLoS One. 2017 Sep 8;12(9):e0184455. doi: 10.1371/journal.pone.0184455 (PMC5590955; doi:10.1371/journal.pone.0184455)

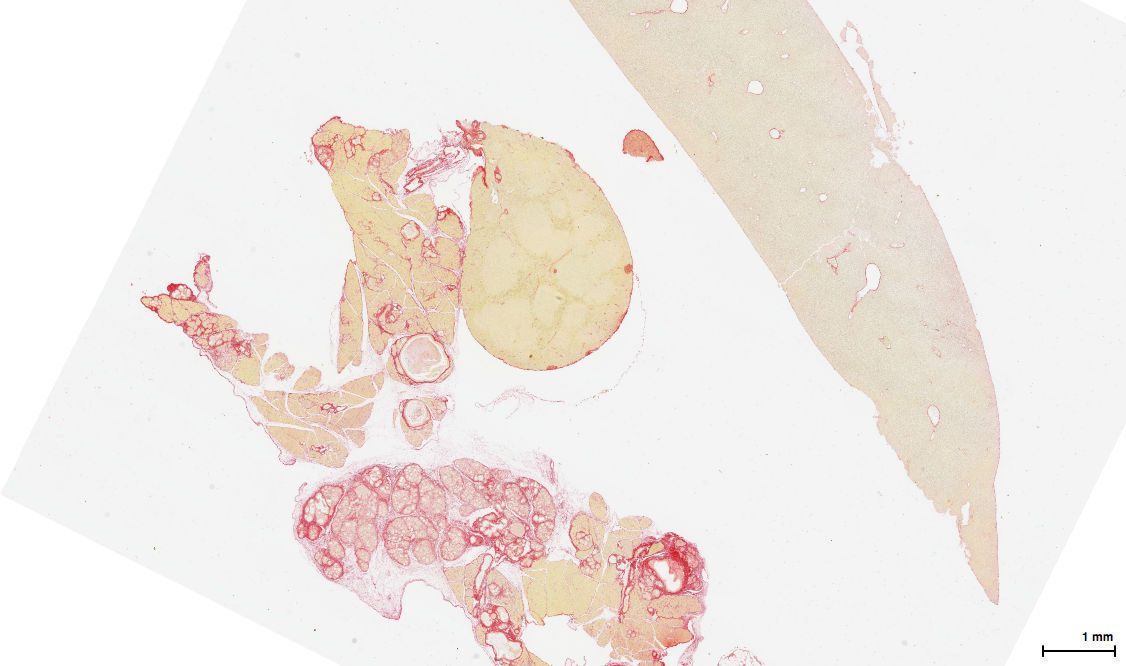

Supplement: S1 Files — (ZIP) [file pone.0184455.s003.zip › Fig6/Fig6B/KC_CD_3747_0.6x.jpeg]

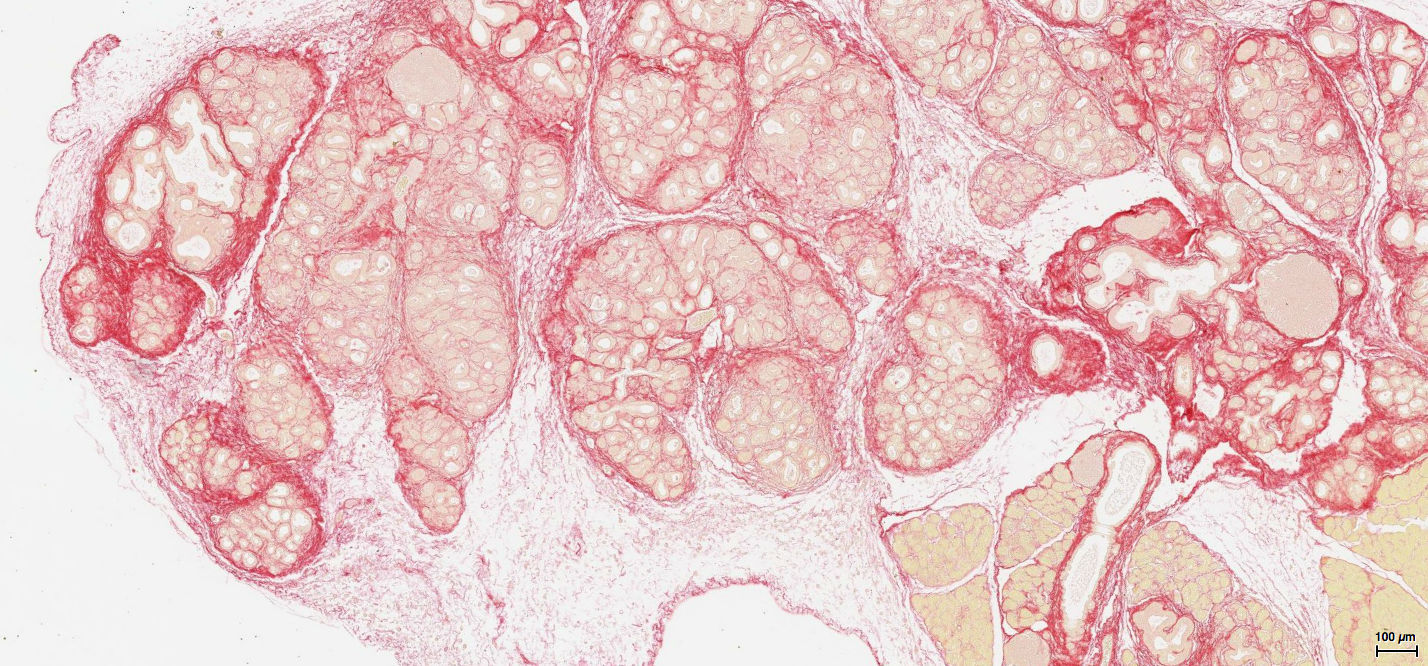

Supplement: S1 Files — (ZIP) [file pone.0184455.s003.zip › Fig6/Fig6B/KC_CD_3747_4x.jpeg]

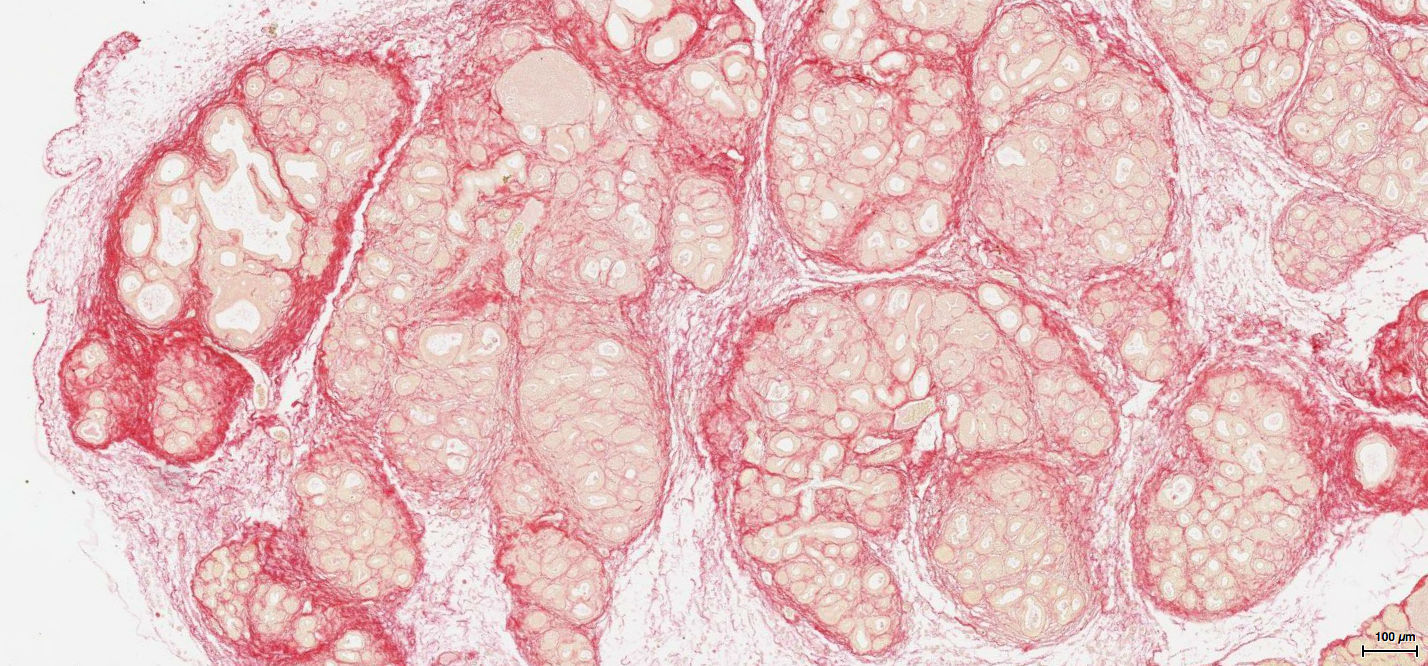

Supplement: S1 Files — (ZIP) [file pone.0184455.s003.zip › Fig6/Fig6B/KC_CD_3747_5x.jpeg]

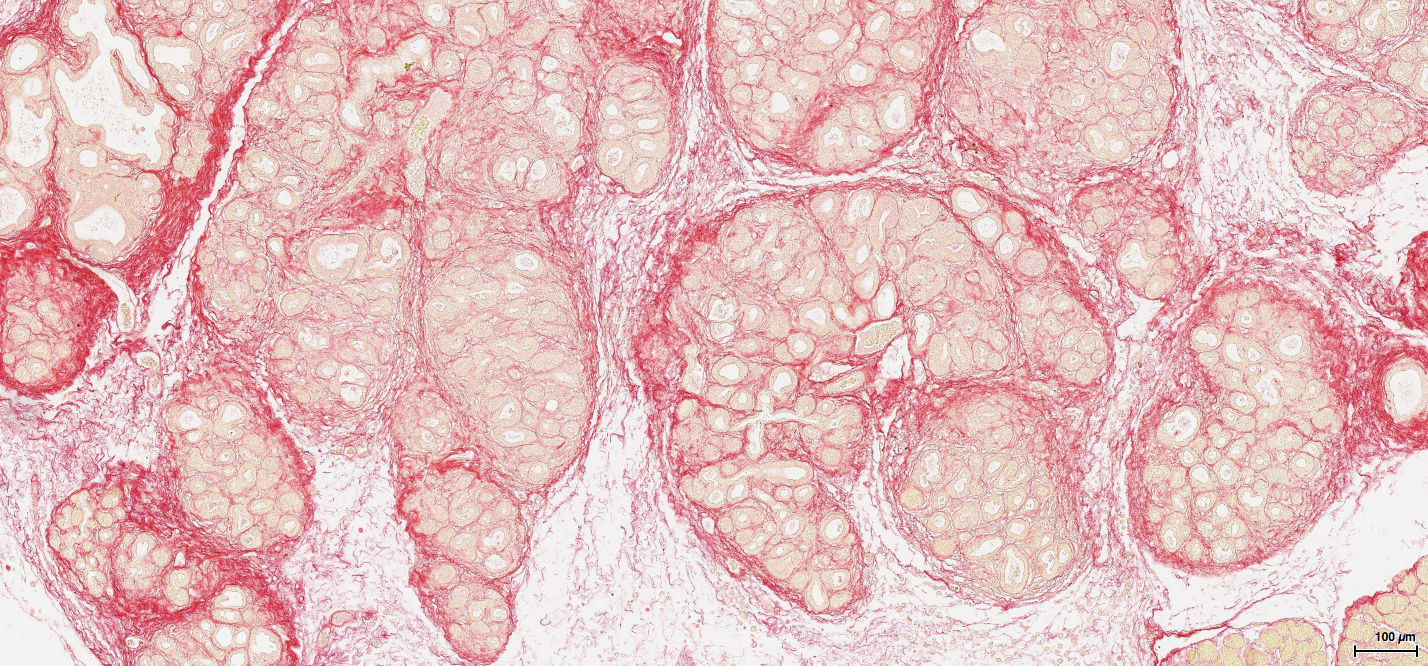

Supplement: S1 Files — (ZIP) [file pone.0184455.s003.zip › Fig6/Fig6B/KC_CD_3747_6.4x.jpeg]

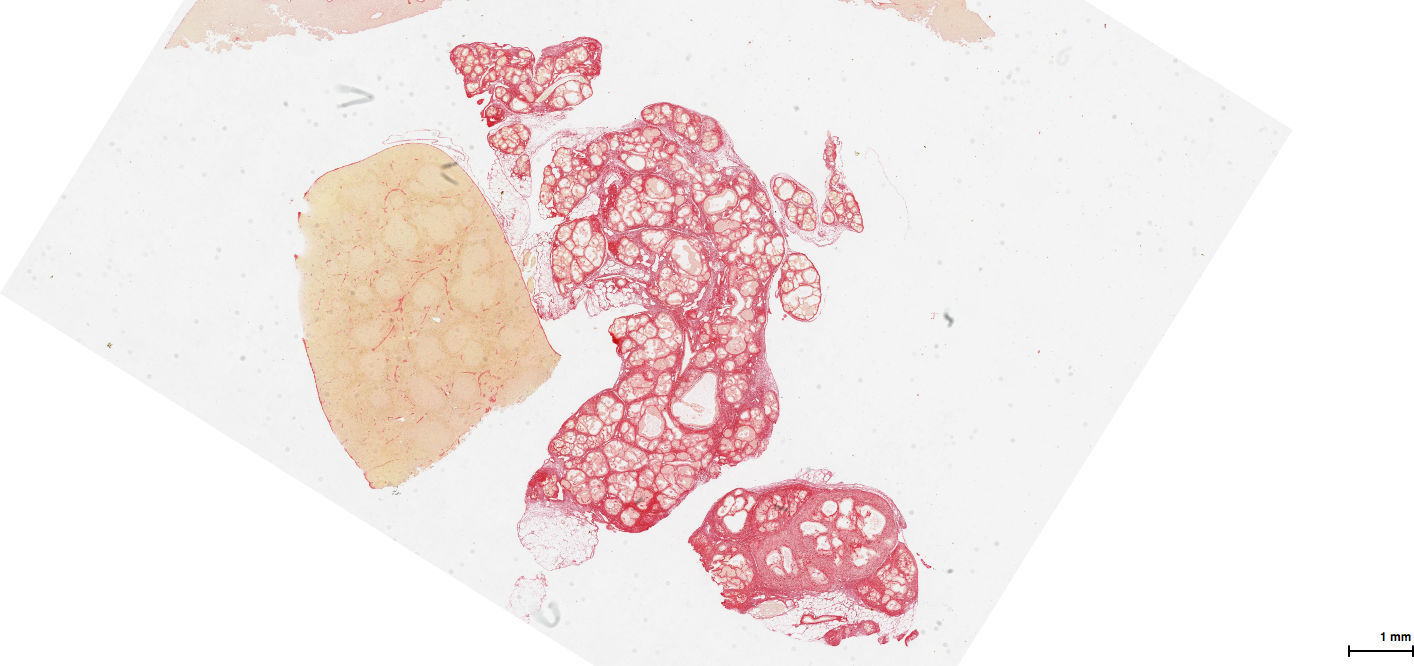

Supplement: S1 Files — (ZIP) [file pone.0184455.s003.zip › Fig6/Fig6B/KC_HF_3637_0.63x.jpeg]

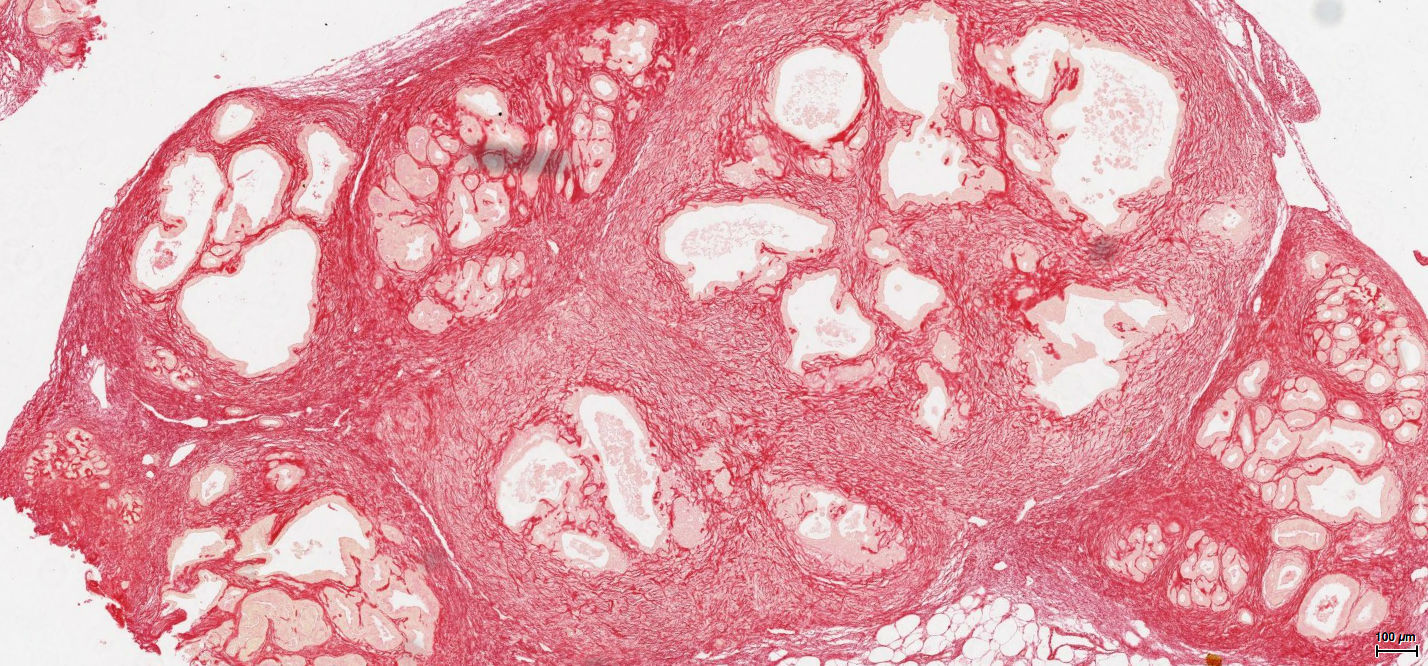

Supplement: S1 Files — (ZIP) [file pone.0184455.s003.zip › Fig6/Fig6B/KC_HF_3637_4x.jpeg]

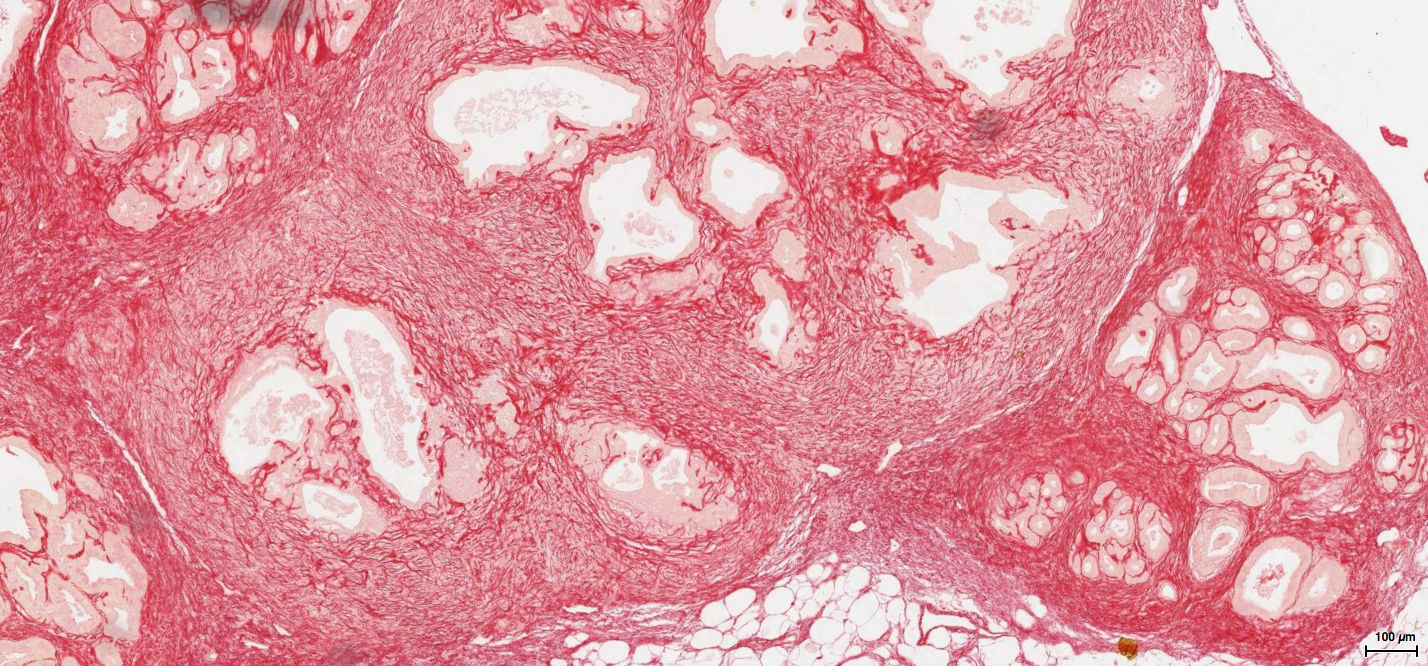

Supplement: S1 Files — (ZIP) [file pone.0184455.s003.zip › Fig6/Fig6B/KC_HF_3637_5x.jpeg]

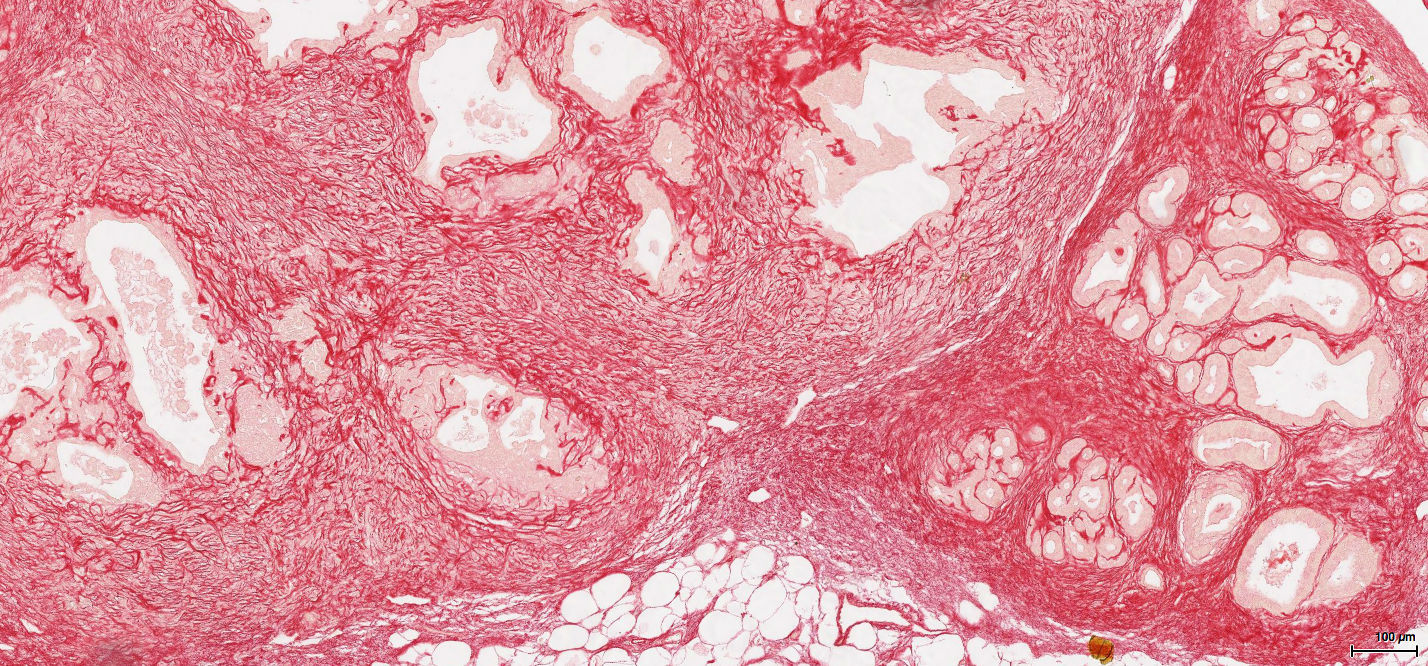

Supplement: S1 Files — (ZIP) [file pone.0184455.s003.zip › Fig6/Fig6B/KC_HF_3637_6.40x_bis.jpeg]

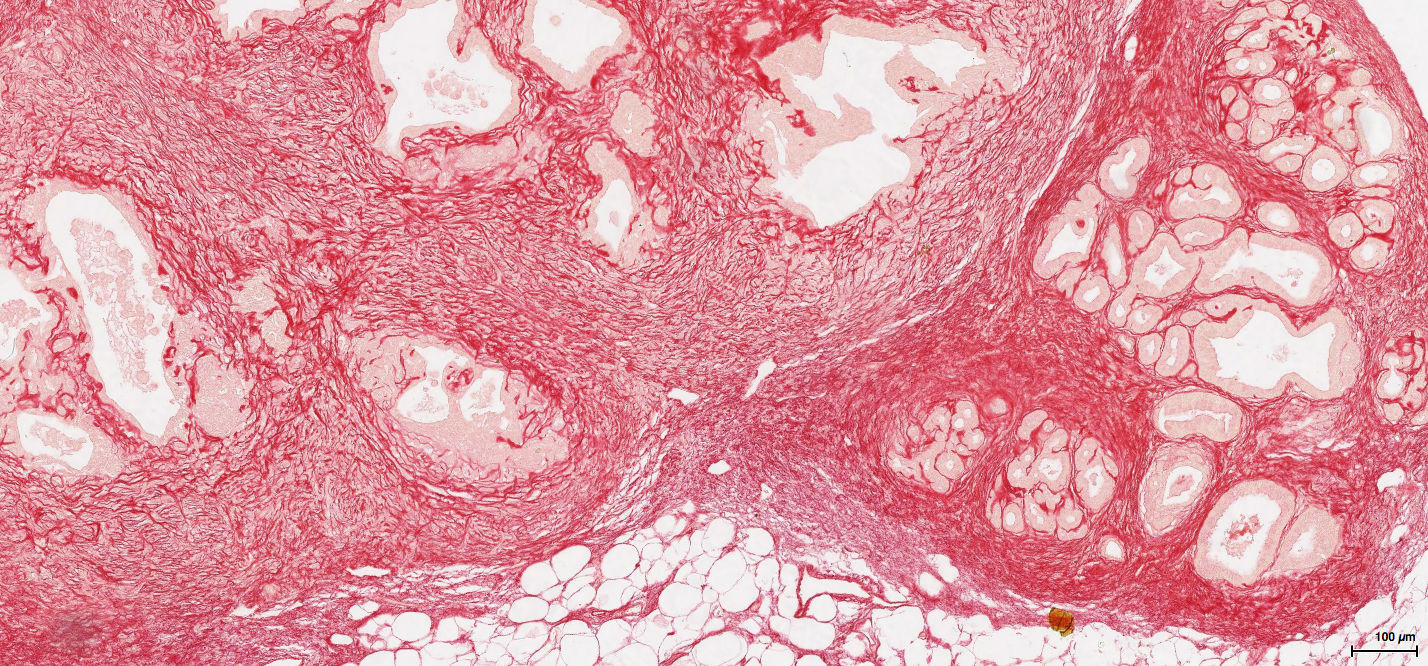

Supplement: S1 Files — (ZIP) [file pone.0184455.s003.zip › Fig6/Fig6B/KC_HF_3637_6.40x_bis2.jpeg]

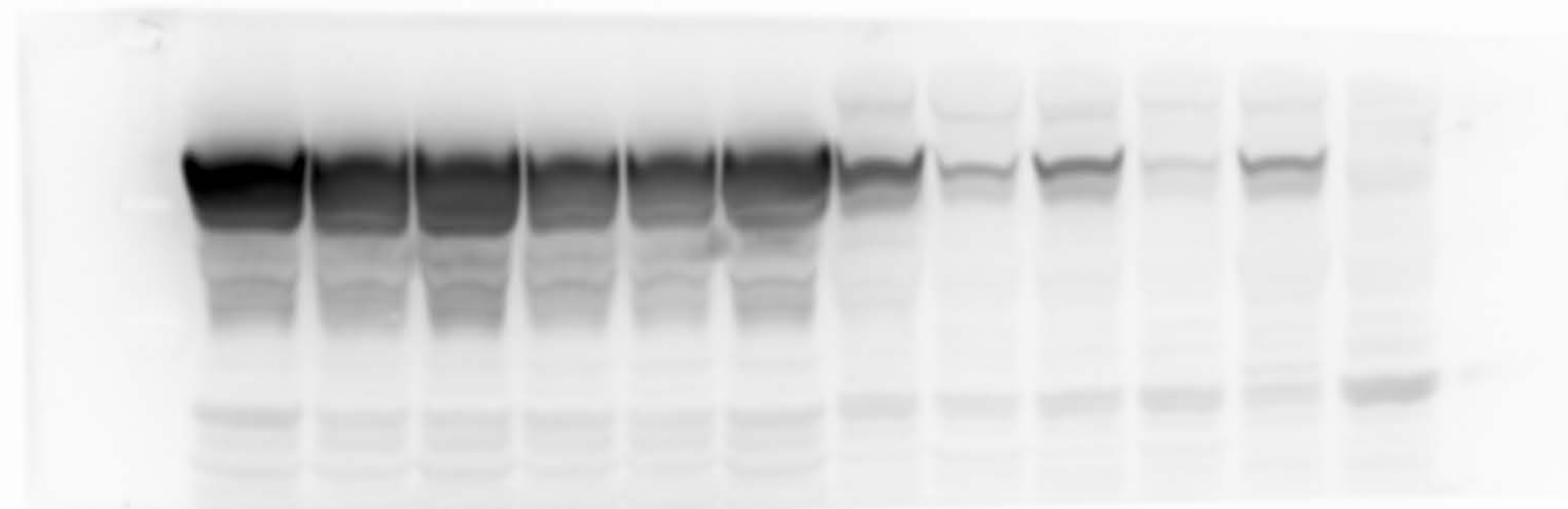

Supplement: S1 Files — (ZIP) [file pone.0184455.s003.zip › Fig6/Fig6C/Amylase mouse tissue WT KC CD HF 09162015.pdf]

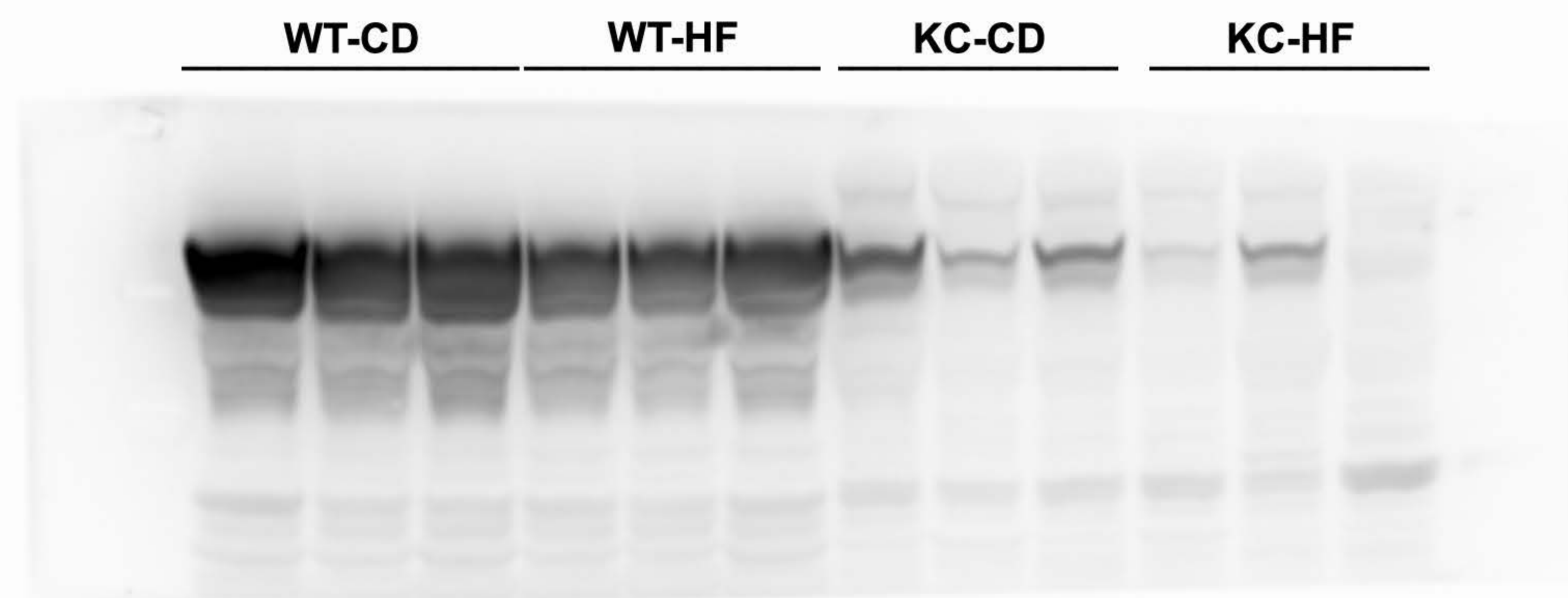

Supplement: S1 Files — (ZIP) [file pone.0184455.s003.zip › Fig6/Fig6C/Amylase mouse tissue WT KC CD HF 09162015_labeled.pdf]

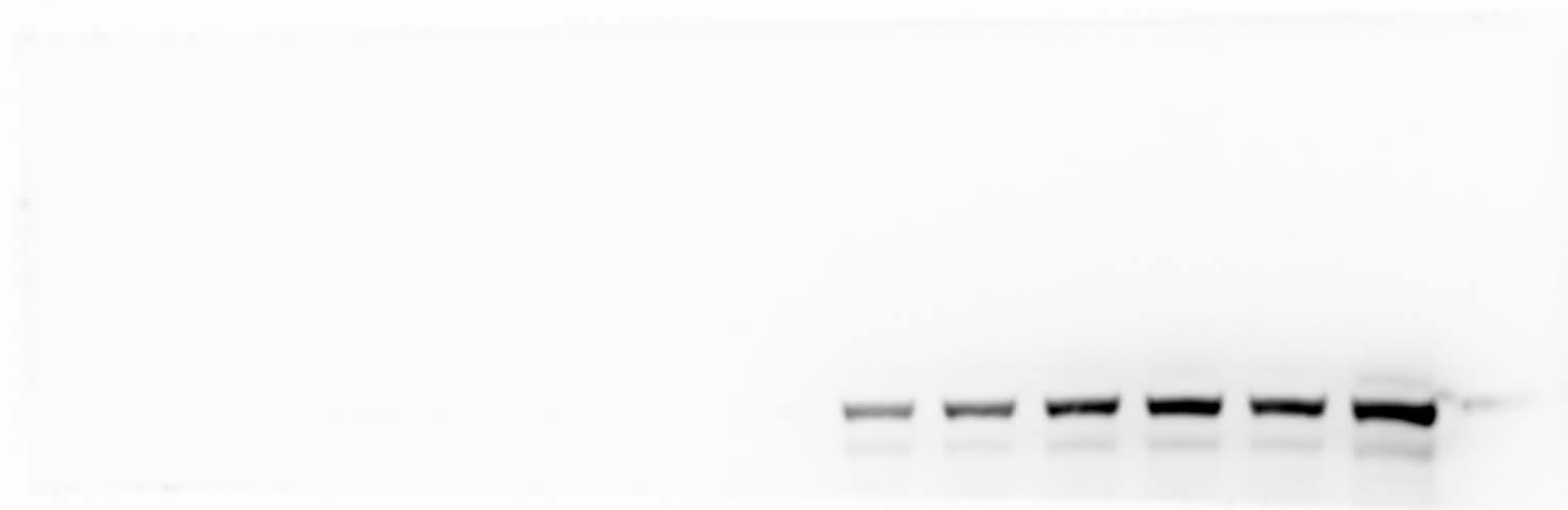

Supplement: S1 Files — (ZIP) [file pone.0184455.s003.zip › Fig6/Fig6C/Cdh11 mouse tissue WT KC CD HF 09012015.pdf]

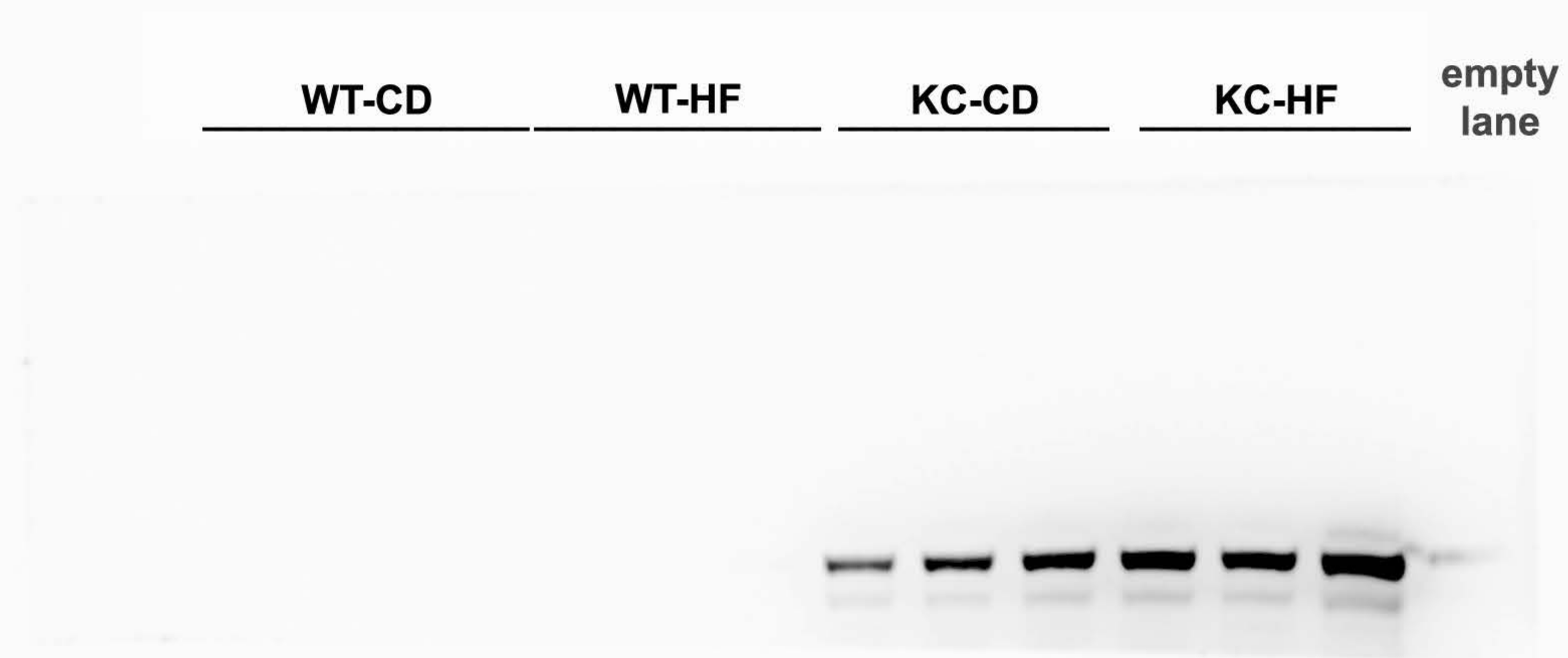

Supplement: S1 Files — (ZIP) [file pone.0184455.s003.zip › Fig6/Fig6C/Cdh11 mouse tissue WT KC CD HF 09012015_labeled.pdf]

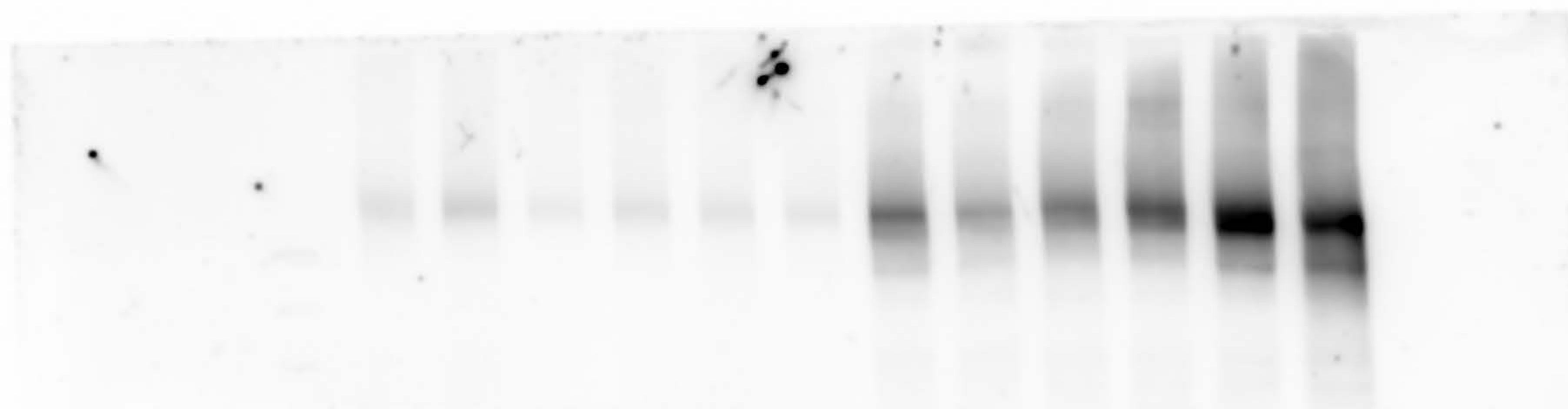

Supplement: S1 Files — (ZIP) [file pone.0184455.s003.zip › Fig6/Fig6C/fibronectin mouse tissue WT KC CD HF 09042015.pdf]

**WT-CD**

**WT-HF**

**KC-CD**

**KC-HF**

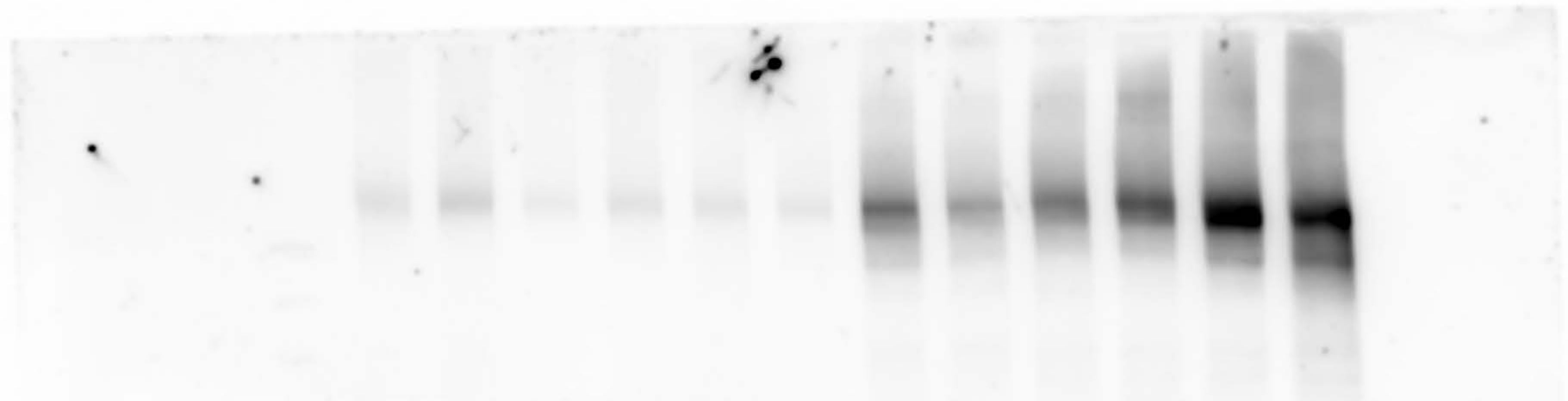

Supplement: S1 Files — (ZIP) [file pone.0184455.s003.zip › Fig6/Fig6C/fibronectin mouse tissue WT KC CD HF 09042015_labeled.pdf]

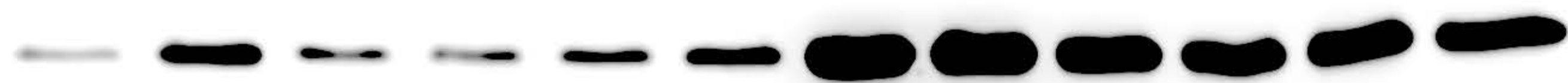

Supplement: S1 Files — (ZIP) [file pone.0184455.s003.zip › Fig6/Fig6C/GAPDH mouse tissue WT KC CD HF 09282015b.pdf]

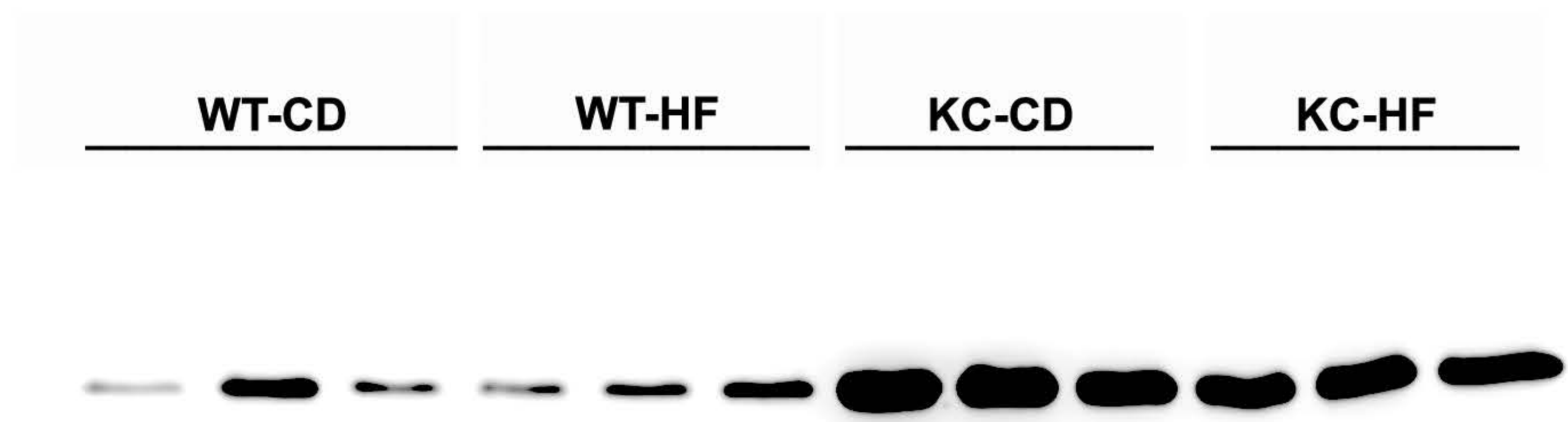

Supplement: S1 Files — (ZIP) [file pone.0184455.s003.zip › Fig6/Fig6C/GAPDH mouse tissue WT KC CD HF 09282015b_labeled.pdf]

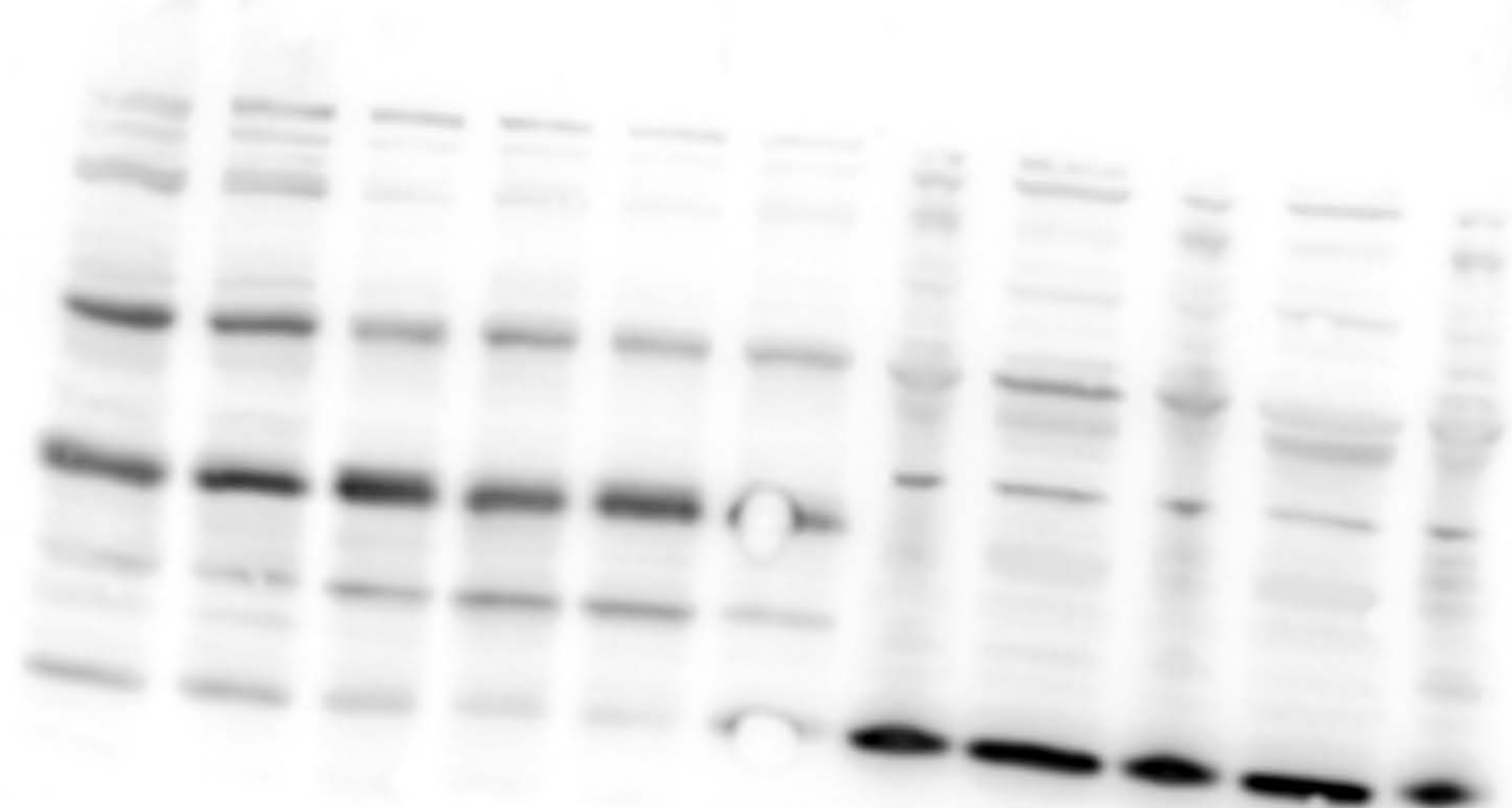

Supplement: S1 Files — (ZIP) [file pone.0184455.s003.zip › Fig6/Fig6C/P4HA1 mouse tissue WT KC CD HF 12162015.pdf]

KC-HF

KC-CD

WT-HF

WT-CD

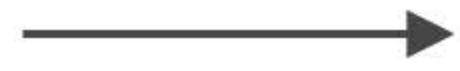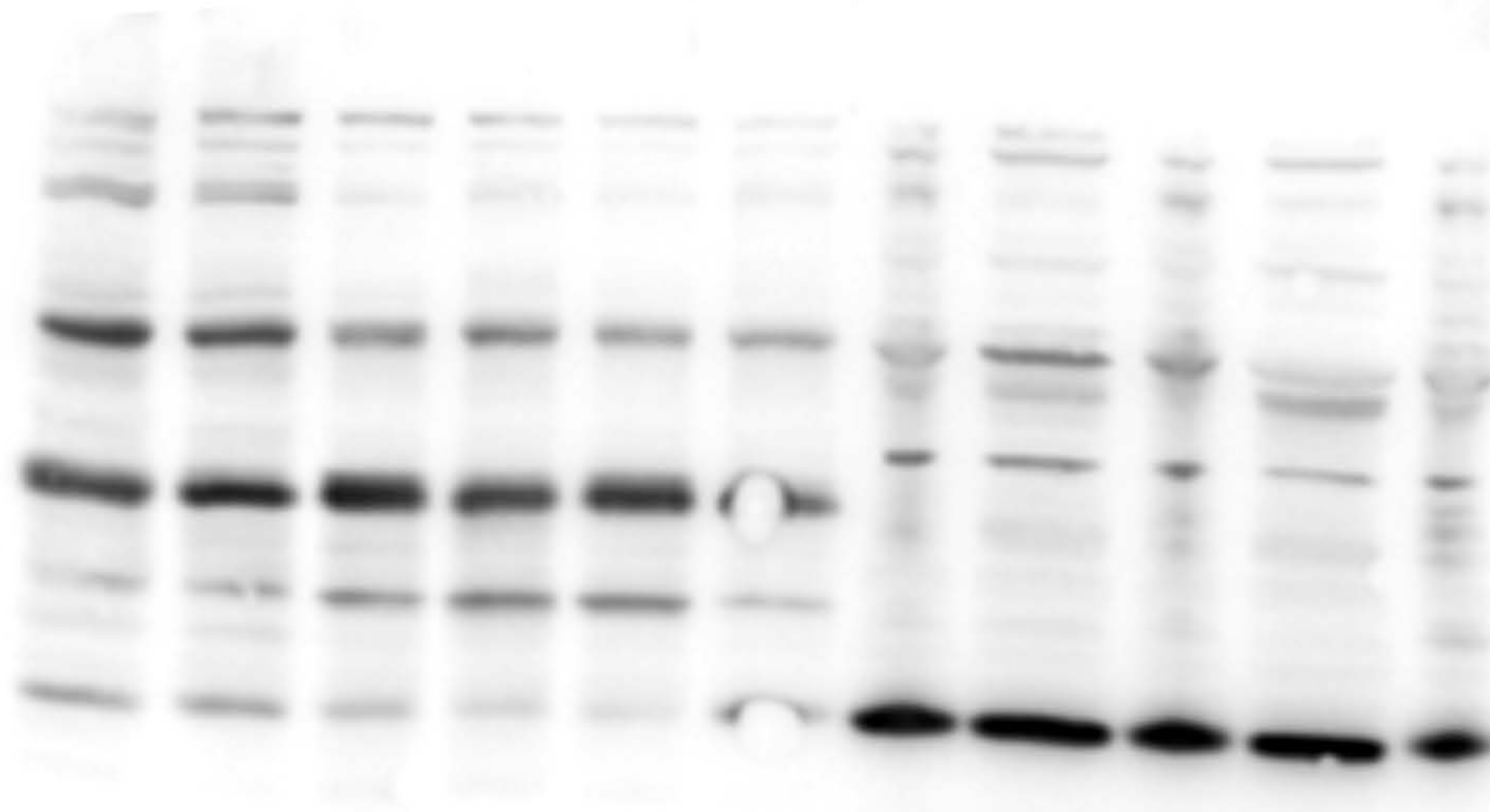

Supplement: S1 Files — (ZIP) [file pone.0184455.s003.zip › Fig6/Fig6C/P4HA1 mouse tissue WT KC CD HF 12162015_labeled.pdf]

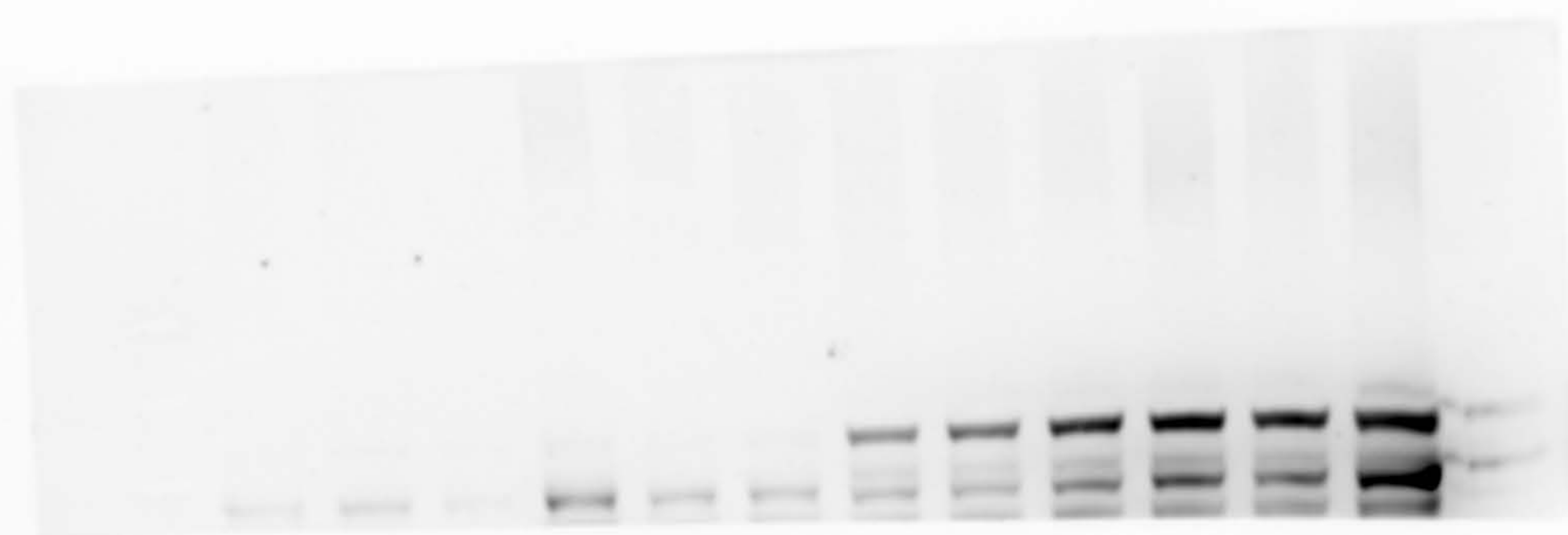

Supplement: S1 Files — (ZIP) [file pone.0184455.s003.zip › Fig6/Fig6C/pSTAT3 mouse tissue WT KC CD HF 09042015 bottom band.pdf]

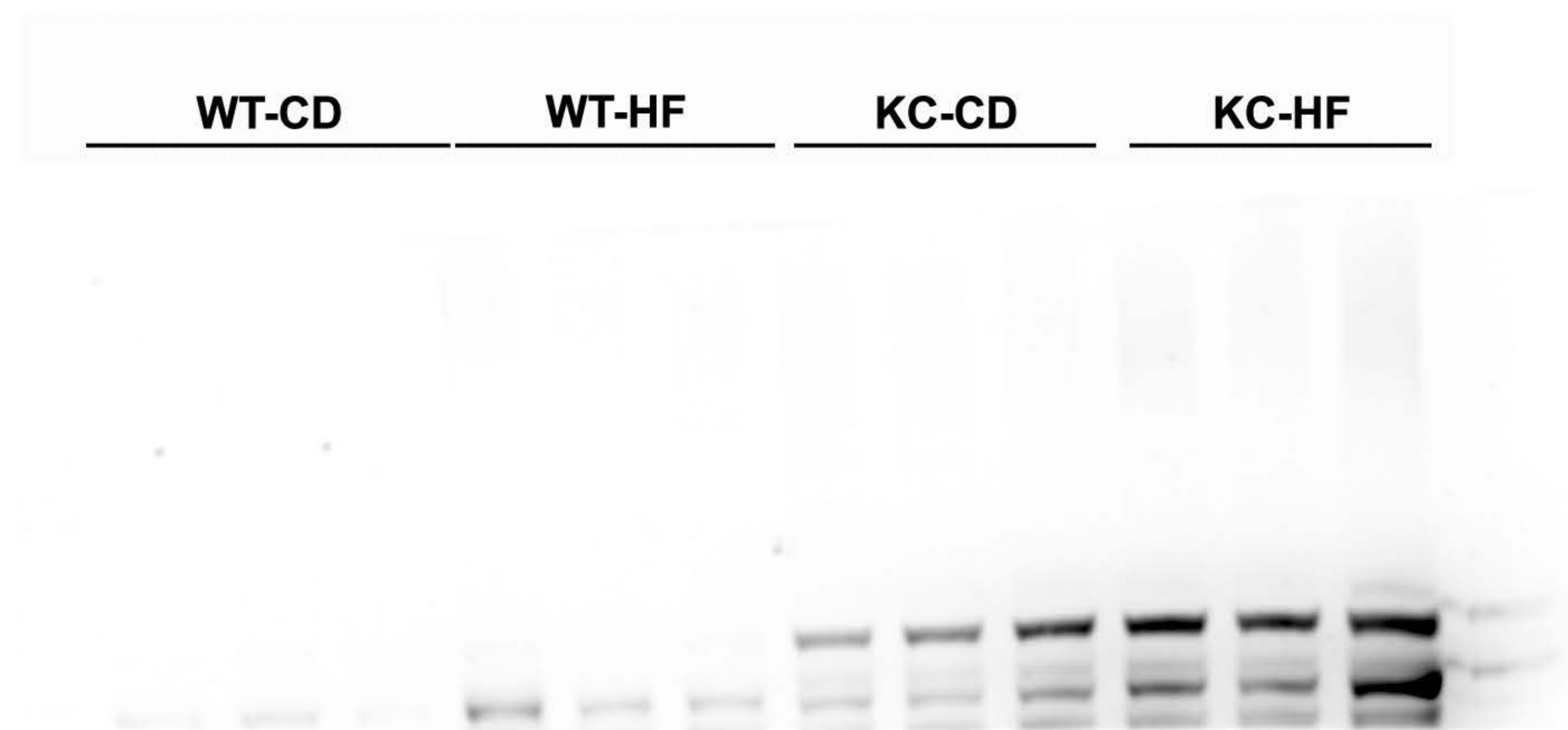

Supplement: S1 Files — (ZIP) [file pone.0184455.s003.zip › Fig6/Fig6C/pSTAT3 mouse tissue WT KC CD HF 09042015 bottom band_labeled.pdf]

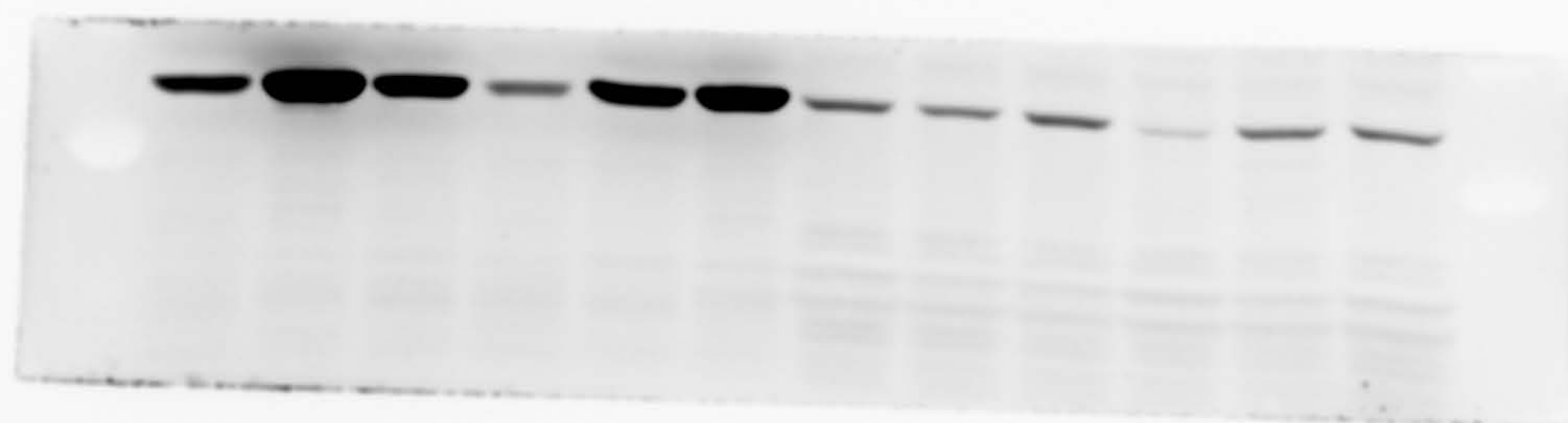

Supplement: S1 Files — (ZIP) [file pone.0184455.s003.zip › Fig6/Fig6C/SMA mouse tissue WT KC CD HF 092315b.pdf]

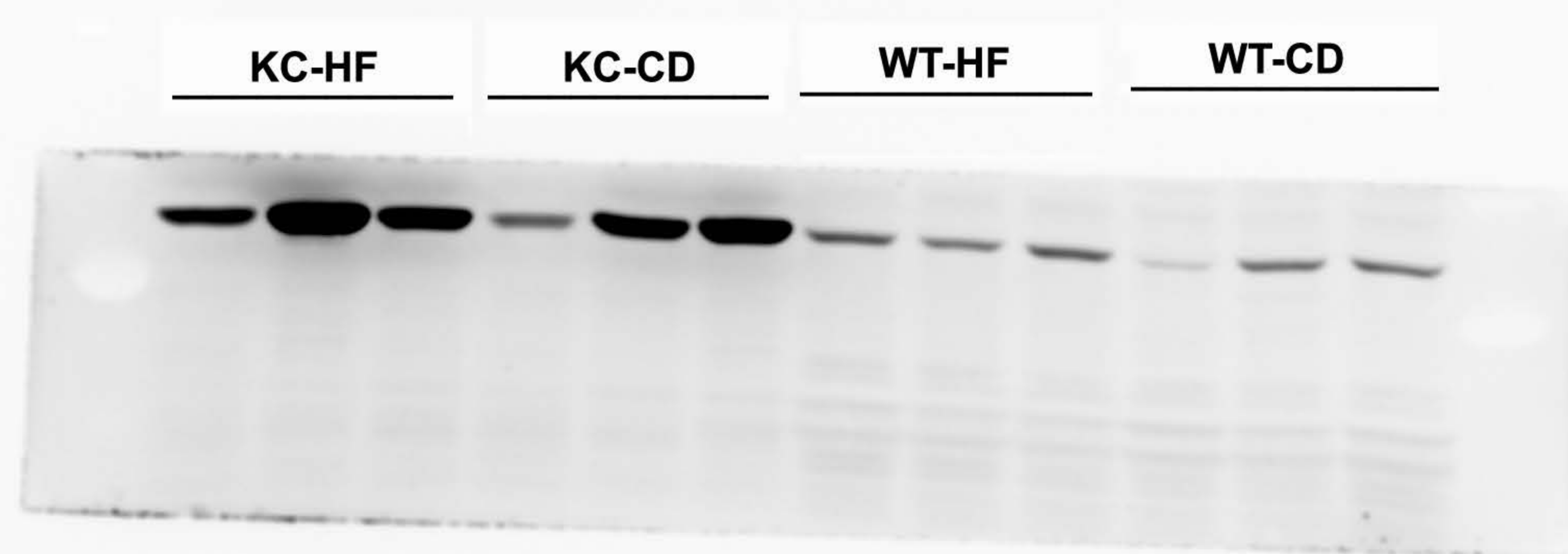

Supplement: S1 Files — (ZIP) [file pone.0184455.s003.zip › Fig6/Fig6C/SMA mouse tissue WT KC CD HF 092315b_labeled.pdf]

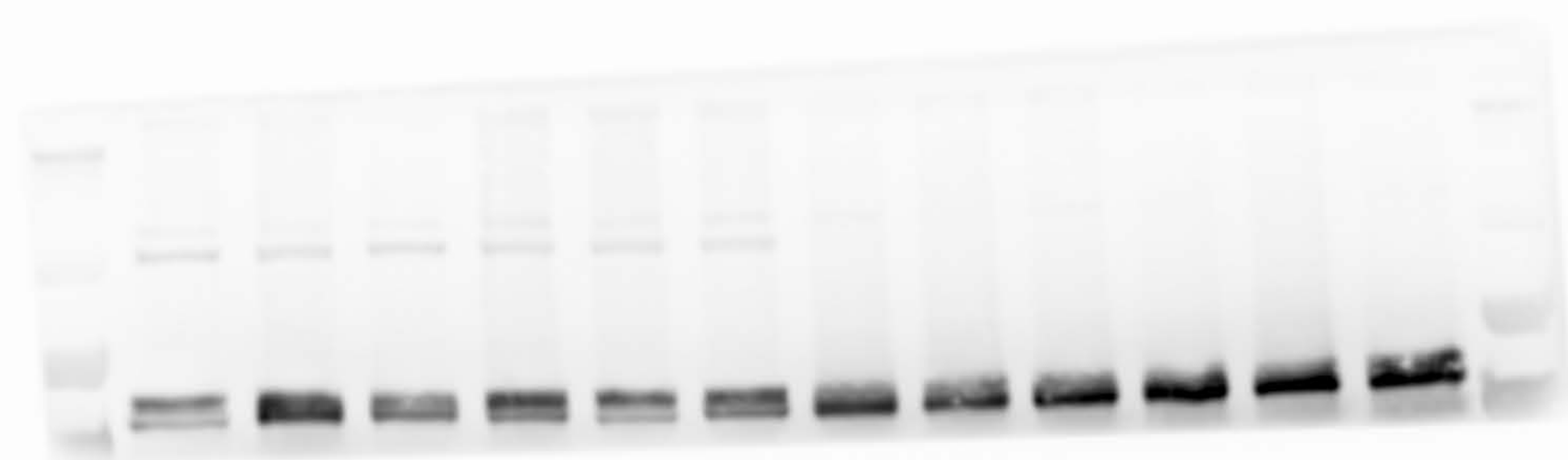

Supplement: S1 Files — (ZIP) [file pone.0184455.s003.zip › Fig6/Fig6C/STAT3 mouse tissue WT KC CD HF 092815b.pdf]

**WT-CD**

**WT-HF**

**KC-CD**

**KC-HF**

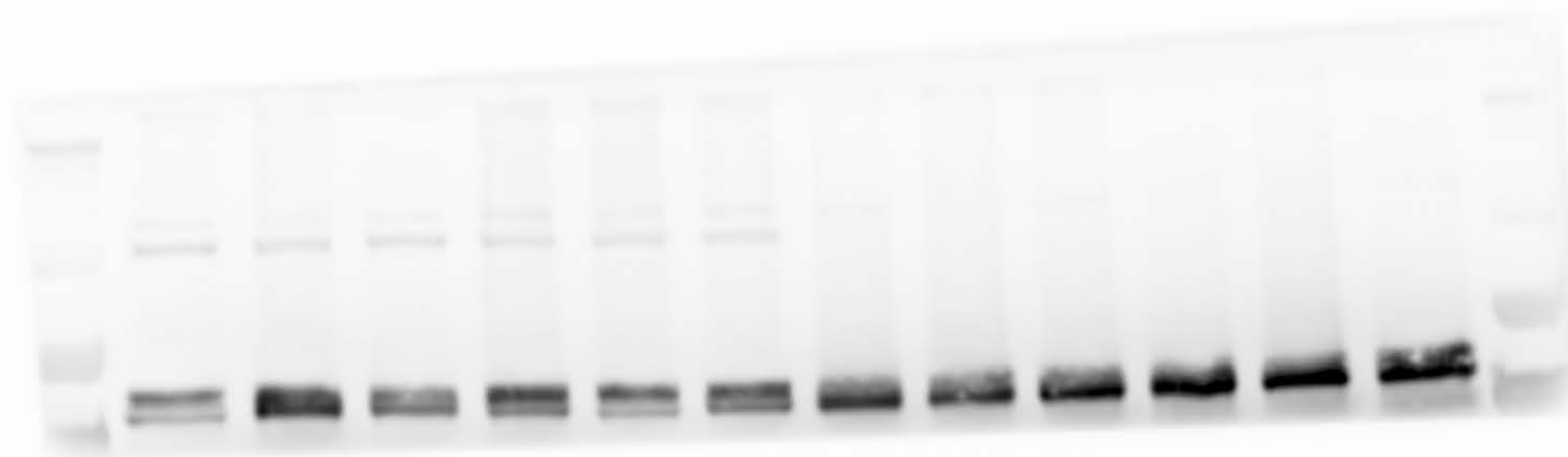

Supplement: S1 Files — (ZIP) [file pone.0184455.s003.zip › Fig6/Fig6C/STAT3 mouse tissue WT KC CD HF 092815b_labeled.pdf]

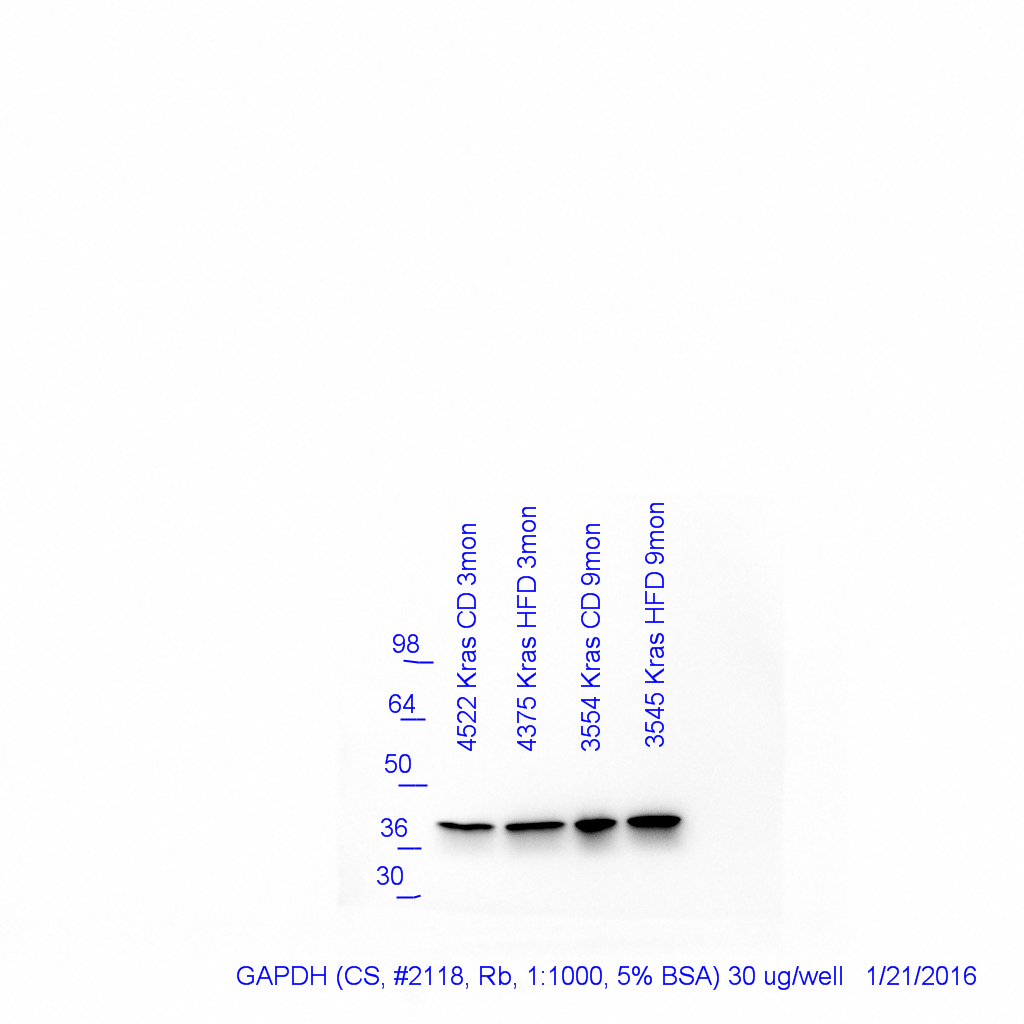

Supplement: S1 Files — (ZIP) [file pone.0184455.s003.zip › Fig7/WB GAPDH HFD.tif]

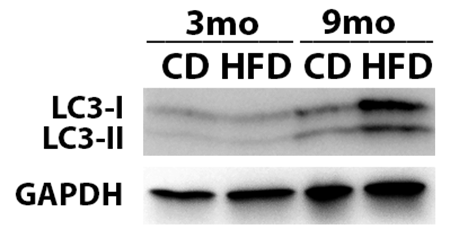

Supplement: S1 Files — (ZIP) [file pone.0184455.s003.zip › Fig7/WB HFD LC3 GAPDH.tif]

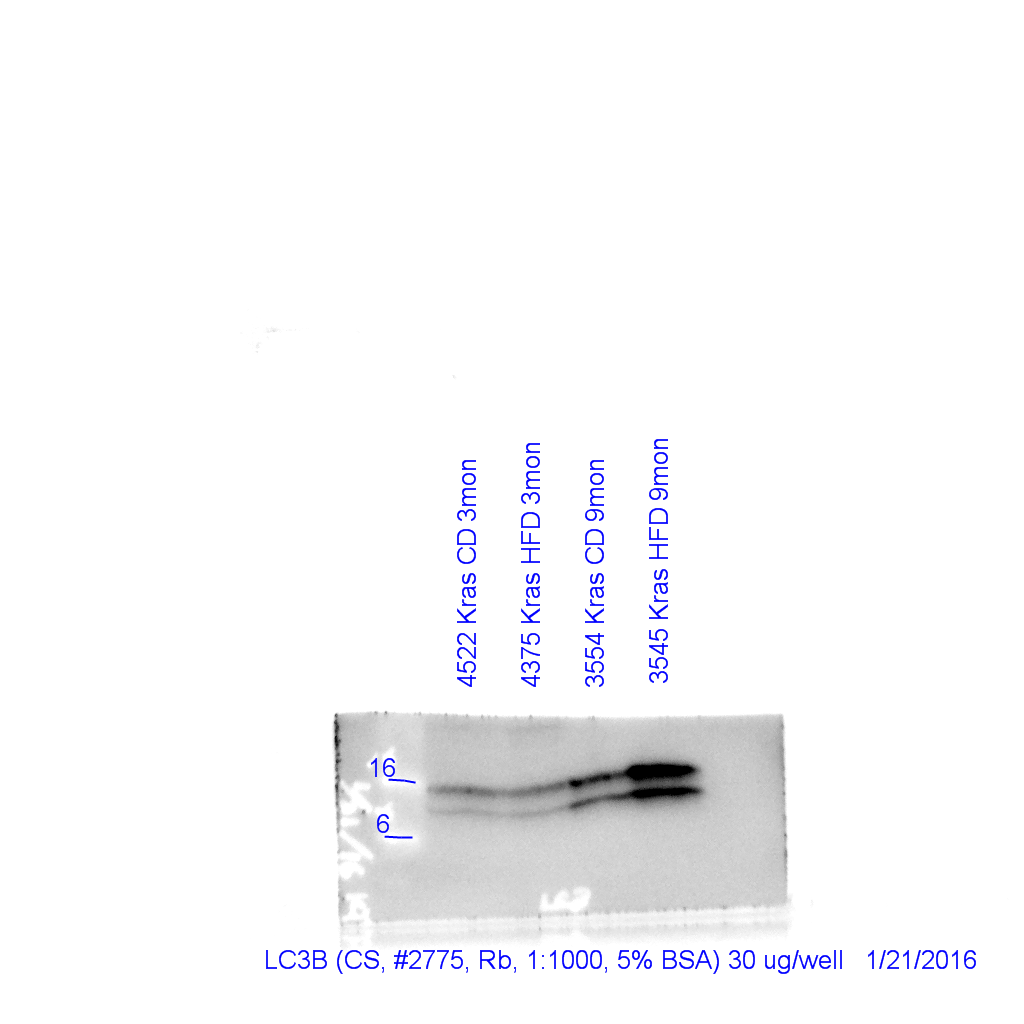

Supplement: S1 Files — (ZIP) [file pone.0184455.s003.zip › Fig7/WB LC3 HFD.tif]
